# Wikis and Collaborative Writing Applications in Health Care: A Scoping Review

**DOI:** 10.2196/jmir.2787

**Published:** 2013-10-08

**Authors:** Patrick M Archambault, Tom H van de Belt, Francisco J Grajales III, Marjan J Faber, Craig E Kuziemsky, Susie Gagnon, Andrea Bilodeau, Simon Rioux, Willianne LDM Nelen, Marie-Pierre Gagnon, Alexis F Turgeon, Karine Aubin, Irving Gold, Julien Poitras, Gunther Eysenbach, Jan AM Kremer, France Légaré

**Affiliations:** ^1^Département de médecine familiale et médecine d'urgenceUniversité LavalQuébec, QCCanada; ^2^Centre de recherche du Centre hospitalier affilié universitaire de LévisCentre de santé et de services sociaux Alphonse-Desjardins (Centre hospitalier affilié universitaire de Lévis)Lévis, QCCanada; ^3^Division de soins intensifsUniversité LavalQuébec, QCCanada; ^4^Centre de recherche du CHU de QuébecAxe Santé des populations - Pratiques optimales en santé, Traumatologie – Urgence – Soins IntensifsQuébec, QCCanada; ^5^Department of obstetrics and gynecologyRadboud University Nijmegen Medical CentreNijmegenNetherlands; ^6^Radboud REshape and Innovation CenterRadboud University Nijmegen Medical CentreNijmegenNetherlands; ^7^Social Media Working GroupInternational Medical Informatics AssociationGenevaSwitzerland; ^8^eHealth Strategy OfficeFaculty of MedicineUniversity of British ColumbiaVancouver, BCCanada; ^9^Scientific Institute for Quality of HealthcareRadboud University Nijmegen Medical CentreNijmegenNetherlands; ^10^Telfer School of ManagementUniversity of OttawaOttawa, ONCanada; ^11^Faculté des sciences infirmièresUniversité LavalQuébec, QCCanada; ^12^Division de soins intensifsDépartement d'anesthésiologieUniversité LavalQuébec, QCCanada; ^13^Université du Québec à RimouskiCampus de LévisLévis, QCCanada; ^14^Association of Faculties of Medicine of CanadaOttawa, ONCanada; ^15^Centre de santé et de services sociaux Alphonse-Desjardins (Centre hospitalier affilié universitaire de Lévis)Lévis, QCCanada; ^16^University Health NetworkCentre for Global EHealth Innovation & Techna InstituteToronto, ONCanada; ^17^Institute for Health Policy, Management, and EvaluationUniversity of TorontoToronto, ONCanada; ^18^JMIR Publications Inc.Toronto, ONCanada; ^19^Canada Research Chair in Implementation of Shared Decision Making in Primary CareQuébec, QCCanada

**Keywords:** collaborative writing applications, collaborative authoring, knowledge management, crowdsourcing, medical informatics, ehealth, Internet, Wiki, Wikipedia, Google Docs, Google Knol, Web 2.0, knowledge translation, evidence-based medicine, participatory med

## Abstract

**Background:**

Collaborative writing applications (eg, wikis and Google Documents) hold the potential to improve the use of evidence in both public health and health care. The rapid rise in their use has created the need for a systematic synthesis of the evidence of their impact as knowledge translation (KT) tools in the health care sector and for an inventory of the factors that affect their use.

**Objective:**

Through the Levac six-stage methodology, a scoping review was undertaken to explore the depth and breadth of evidence about the effective, safe, and ethical use of wikis and collaborative writing applications (CWAs) in health care.

**Methods:**

Multiple strategies were used to locate studies. Seven scientific databases and 6 grey literature sources were queried for articles on wikis and CWAs published between 2001 and September 16, 2011. In total, 4436 citations and 1921 grey literature items were screened. Two reviewers independently reviewed citations, selected eligible studies, and extracted data using a standardized form. We included any paper presenting qualitative or quantitative empirical evidence concerning health care and CWAs. We defined a CWA as any technology that enables the joint and simultaneous editing of a webpage or an online document by many end users. We performed qualitative content analysis to identify the factors that affect the use of CWAs using the Gagnon framework and their effects on health care using the Donabedian framework.

**Results:**

Of the 111 studies included, 4 were experimental, 5 quasi-experimental, 5 observational, 52 case studies, 23 surveys about wiki use, and 22 descriptive studies about the quality of information in wikis. We classified them by theme: patterns of use of CWAs (n=26), quality of information in existing CWAs (n=25), and CWAs as KT tools (n=73). A high prevalence of CWA use (ie, more than 50%) is reported in 58% (7/12) of surveys conducted with health care professionals and students. However, we found only one longitudinal study showing that CWA use is increasing in health care. Moreover, contribution rates remain low and the quality of information contained in different CWAs needs improvement. We identified 48 barriers and 91 facilitators in 4 major themes (factors related to the CWA, users’ knowledge and attitude towards CWAs, human environment, and organizational environment). We also found 57 positive and 23 negative effects that we classified into processes and outcomes.

**Conclusions:**

Although we found some experimental and quasi-experimental studies of the effectiveness and safety of CWAs as educational and KT interventions, the vast majority of included studies were observational case studies about CWAs being used by health professionals and patients. More primary research is needed to find ways to address the different barriers to their use and to make these applications more useful for different stakeholders.

## Introduction

Health care decision makers—providers, patients, managers, and policy makers—are failing to use research evidence to inform their decisions [1]. By involving knowledge users in the creation and dissemination of knowledge [2], social media—highly accessible, Web-based, interactive vehicles of communication—have the potential to empower users to apply knowledge in practice. Acknowledging this potential and recognizing that social media capitalizes on the free and open access to information, scientists, opinion leaders, and patient advocates have called for research to determine whether social media can equip decision-making constituencies to improve health care delivery [3,4] decrease its costs [2,5,6], accelerate knowledge discovery [7-11], and improve access to knowledge within developing countries [4,12-17].

Collaborative writing applications (CWAs) [18,19] are a category of social media that has surged in popularity in recent years, including within the health care sector [2,6,18,20]. CWAs consist of software that allows users to create online content that anyone who has access can edit or supplement [21]. With these contributions, CWAs can become rich multimodal communication tools enriched with hyperlinks, images, videos, and audio. For example, Internet users have turned to wikis [22,23] to produce a Wikipedia entry on the Global Plan to Stop Tuberculosis [4]; to Google Knol [24,25] to exchange research on influenza at the Public Library of Science [26]; and to Google Docs [19,27] to review the literature on emergency medicine [28,29]. Although now defunct, Google Knol was a Google project that aimed to include user-written articles on a range of topics that could be edited only if the original authors gave access to editing the text. CWAs can also be classified based on who has access. There are open or public CWAs such as Wikipedia, which can be edited by anyone in the world and can also be seen by anyone. There are also partially public CWAs, which can be seen by anyone, but can be edited only by certain members of a restricted community (eg, Ganfyd [30]). There are also closed or private CWAs, part of central knowledge management systems (eg, Intelink [31]) or online learning systems (eg, Blackboard [32]), which are edited by members of the institution and are visible only to members of the institution.

Among the types of CWAs, wikis and its most famous representative—Wikipedia—are perhaps the most popular. Wikipedia is an online encyclopedia whose medical articles are viewed about 150 million times per month and exist in 271 languages [4]. Moreover, readership of Wikipedia’s medical content is continuing to increase [33]. New wikis have appeared in all fields of health care [18,28,34-41], and studies of developed countries report 70% of junior physicians using Wikipedia weekly [42]. Patients use wikis to share their experiences [43] and to find information [4]. The Canadian Agency for Drugs and Technologies in Health is exploring the use of wikis to update knowledge syntheses [44-46]; the United States’ National Institutes of Health is training its scientists in editing them [47,48]; and the World Health Organization is using a wiki format to update the International Classification of Diseases [49]. In addition, academic institutions have started using wikis to train health professionals [18,22,32,50-54]. Wikis have come to exemplify social media’s tremendous promise to enable health professionals, patients, and policy makers to implement evidence-based practice at remarkably low cost [5,28,29,55,56]. In doing so, they could contribute to improving the health of millions of people around the world [4,13].

 However, questions remain about the safety [57-59], reliability [60-64], lack of traditional authorship [65,66], and the legal implications for decision making [67,68] regarding the use of CWAs in health care. Researchers question clinicians’ intentions to use the applications in their practice [28] and to contribute knowledge collaboratively [4,29,69]. Furthermore, it is unknown how CWAs can enhance the delivery of health care (eg, by empowering patients in decision making [70,71], by improving health care communication and education [18,27,32,72,73,74,75]), and benefiting health in developing countries [4,76]. While researchers have conducted systematic reviews on Internet and communication technologies (ICTs) [77,78] social media in health care [79-84] and research on Wikipedia in general [85], none have specifically focused on wikis and CWAs in health care. Not all social media share the same mechanisms of action [21], therefore examining CWAs in health care is important. The overarching goal of this project was to explore the depth and breadth of evidence about the effective, safe, and ethical use of wikis and CWAs in health care. We conducted a scoping review with the following specific objectives: (1) to map the literature on the use of wikis and other CWAs in health care, (2) to compare the applications’ features by investigating how they were used in collaborative writing projects, (3) to synthesize the applications’ positive and negative effects as knowledge translation interventions in health care, (4) to inventory the barriers and facilitators that affect how they influence health care delivery, and (5) to produce a research agenda delimiting areas where further knowledge synthesis is needed and where more primary research remains to be done.

## Methods

### Overview

A detailed description of our peer-reviewed research protocol and conceptual framework can be found elsewhere [86]. This review was planned, conducted, and reported in adherence to standards of quality for scoping reviews [87,88]. A summary of our six-stage methodology follows.

### Stage 1: Identifying the Research Question

Our research question was developed by consulting a group of knowledge users to determine their needs and questions about using collaborative writing applications for knowledge translation. We defined “collaborative writing applications” as a category of social media that enables the joint and simultaneous editing of a webpage or an online document by many end users (eg, wikis, Wikipedia, Google Knol, Google Docs, Google Sites) [21]. The participants targeted by this scoping review were health care stakeholders.

### Stage 2: Identifying Studies and Grey Literature

Seven scientific databases (Cochrane Library, PubMed, EMBASE, CINAHL, PsycINFO, ERIC, ProQuest Dissertations and Theses) were searched systematically for the period covering January 1, 2001 (Wikipedia’s inaugural year), to September 16, 2011. Our search strategy was peer-reviewed using the PRESS criteria [89]. The following keywords were used and adapted to each database: “wiki”, “wikis”, “Web 2.0”, “social media”, “Google Knol”, “Google Docs”, and “collaborative writing applications” (see Table 1).

We did not exclude any citations based on language. In addition, study reference lists; the 2010 and 2011 editions of the Medicine 2.0, WikiSym, and American Medical Informatics Association conference proceedings; clinicaltrials.gov and Open Medicine’s websites; expert consultation (eg, the authors of WikiProject Medicine [4]), OpenSIGLE (before 2005), and the Health Technology Assessment international Vortal were searched. Furthermore, environmental scans of the grey literature indexed by Google, Bing, Yahoo, and Mednar were performed. Finally, via email, Twitter, Mendeley, Google Docs, and a health librarianship page (HLWIKI), we called for the crowdsourcing of studies that could potentially fall within the scope of this review.

**Table 1:** Full search strategy for each database.

Pubmed	Wiki*[All Fields] OR “Web 2.0”[TIAB] OR “Web2.0”[TIAB] OR (google* AND knol) OR (google* AND docs) OR “Social media” [TIAB] OR (Collaborative [tiab] AND writing [tiab]) OR (collaborative technolog*) OR (collaborative software*)
Embase	wiki* OR “collaborative technology” OR “collaborative technologies” OR “collaborative writing” OR “collaborative writings” OR “collaborative software” OR “collaborative softwares” OR “google docs” OR “google knol” OR “ehealth 2.0” OR “health 2.0” OR “e+health 2.0” OR “Web 2.0”
CINAHL	TI (wiki* or “google docs” or “google knol” or “medecine 2.0.” or “Web 2.0” or “collaborative technolog*” or “collaborative writing” or “ehealth” or “e-health” or emedicine or “e-medicine”) OR AB (wiki* or “google docs” or “google knol” or “medecine 2.0.” or “Web 2.0” or “collaborative technolog*” or “collaborative writing” or “ehealth” or “e-health” or emedicine or “e-medicine”)
PsychINFO	(wiki* or “google docs” or “google knol” or “collaborative software” or “collaborative writing” or “collaborative technologies” or “collaborative technology” ):Any Field OR ( “medicine 2.0” or “emedicine” or e-medicine or “health 2.0” or “ehealth” or e-health or “Web 2.0” ):Title OR ( “medicine 2.0” or “emedicine” or e-medicine or “health 2.0” or “ehealth” or e-health or “Web 2.0” ):Abstract
ERIC	((Keywords:wiki* or Keywords: “Web 2.0” or Keywords: “google docs” or Keywords: “google knol” or Keywords: “collaborative technologies” or Keywords: “collaborative technology” or Keywords: “collaborative software” or Keywords: “collaborative writing” or Keywords: “e-health” or Keywords: ehealth) or (Title: wiki* or Title: “Web 2.0” or Title: “google docs” or Title: “google knol” or Title: “collaborative technologies” or Title: “collaborative technology” or Title: “collaborative software” or Title: “collaborative writing” or Title: “e-health” or Title: ehealth) and (Thesaurus Descriptors: “Health services”))
Dissertation abstract & Thesis	Citation & Abstract (wiki* or “health 2.0” or “Web 2.0” or “e-medicine” or emedicine or “google docs” or “google knol” or “collaborative technologies” or “collaborative technology” or “collaborative writing” or “collaborative software”)
Cochrane Library (n=56)	(wiki* or “Web 2.0” or ehealth or “e-health” or “google docs” or “google knol” or “collaborative writing”) in Title, Abstract or Keywords in All Cochrane Library
Google, Bing, and Yahoo (n=1200 in total)	“wiki in health care”; “Google Knol in health care”; “Google Docs in health care”; “collaborative writing applications in health care”

### Stage 3: Selecting Studies

Three teams of 2 reviewers (SR/MF, TB/AB, PA/CK) independently screened titles, abstracts, and grey literature and retained articles that presented empirical data about any CWA applied to the field of health care. In case of disagreements, a third reviewer was consulted (PA, TB, or SR). To reach a high level of agreement, we conducted 4 series of assignments (400 abstracts in total) whereby the screening of a number of studies was followed by a teleconference to reach agreement about which studies to include and to discuss uncertainties. Once consensus was reached for all cases, the remaining studies were coded by the same 3 pairs of screeners (SR/MF, TB/AB, PA/CK). Subsequently, 2 reviewers (TB and PA) conducted another round of screening based on full text studies. As a result, a narrowed definition of health care was applied in order to focus the analysis. Hence, studies that concerned the care of patients were included, and those from the fields of basic medical sciences, the conduct of clinical trials, biomedical library science and medical informatics were excluded.

### Stage 4: Charting the Data

A data-charting form was developed and built into EPPI-Reviewer for the extraction of quantitative and qualitative variables and to facilitate data coding. It was tested and refined by 4 reviewers (PA, CN, ME, CF) using the first 50 studies. Three pairs of 2 reviewers (CN/CF, CN/ME, ME/CF) then independently extracted data from the remaining studies. Disagreements were resolved through discussion with a third reviewer (PA or TB). Using EPPI-Reviewer’s inductive coding function, we extracted all the pre-planned variables described in our published protocol [86].

### Stage 5: Collating, Summarizing, and Reporting Results

#### Themes Overview

We summarized the included studies in a table comparing each of the study’s characteristics. Attempting to present an organized description of the current literature on the use of CWAs in health care, we grouped studies based on purpose. Three emergent themes were the use patterns of CWAs (Theme 1), quality of information found in different CWAs (Theme 2), and CWAs used as knowledge translation interventions (Theme 3). We also added a description of each of the applications’ features (the type of CWA and software used) to examine CWA use among studies (Objective 2).

To compare the different CWA applications identified, a Venn diagram was constructed to situate each application in relation to the others depending on two features: their collaborative writing features and their conversational features. To create the most reliable representation of how different CWAs could be represented in relation to each other, each CWA was assessed by 2 reviewers using a scoring system we created based on a classification proposed by Kaplan et al [21]. We attributed a score of 1-5 to characterize the extent of their collaborative writing features and a score of 1-5 to measure the extent of their conversational features. To design our Venn diagram, we plotted each different CWA on a graph presenting the conversational features score on the x axis and the collaborative writing score on the y axis.

#### Theme 1: Use Patterns of CWAs

Studies whose purpose was to describe the users and the frequency of CWA use were grouped together. We compared each study in a table presenting the population surveyed, the response rate of the population surveyed, the reported results, the prevalence of use, the contribution rate, the time of assessment, and the purpose of CWA use. We also used Eysenbach’s Medicine 2.0 map [2] to illustrate the extent to which the different CWAs described in the included studies involve three major stakeholder groups (consumers/patients, professionals, and researchers).

#### Theme 2: Quality of Information in Different CWAs

We synthesized papers that evaluated the quality of information in CWAs by constructing a table presenting a summary of each evaluation. Three reviewers (PA, TB, SG) assigned a score on a three-point scale based on the original authors’ own recommendations about future use of information contained in the different CWAs. When authors concluded that the information contained within the collaborative writing project was of high quality and that it could be used in medical decision making, we gave the paper a score of 1. When the authors concluded that the information reported was not reliable and should never be used in decision making, a score of 3 was attributed. When authors were uncertain and/or suggested that more research was needed, a score of 2 was given. This score was attributed after discussion between the three reviewers until consensus was achieved.

#### Theme 3: CWAs Used as Knowledge Translation Interventions

##### Positive/Negative Effects

Three reviewers (PA, TB, SG) performed a mixed inductive and deductive thematic analysis of the content coded in Stage 4 to classify and interpret the perceived positive and negative effects related to the use of a CWA. They began by developing a coding scheme using qualitative content analysis, a method whereby reviewers interpreted the data subjectively by classifying and coding data and identifying patterns [90]. Then, they read the data charted in Stage 4 repeatedly to immerse themselves and obtain a broad perspective [91]. Subsequently, using constant comparison methodology [90], they read the coded content by each reviewer in Stage 4, highlighting words that captured the positive or negative effects. A matrix was created to present any positive or negative effect reported in each study. We then assigned these effects specific codes, organized them into broad categories, and developed a tree diagram to organize the categories into a hierarchical structure [92]. We consolidated codes and categories that expressed the same idea into a comprehensive coding scheme that constituted our taxonomy and guided reviewers’ content analysis of the rest of the data. The three reviewers discussed units of text that could not be coded with existing codes and created new codes if necessary.

The Donabedian framework [93] for quality improvement informed the classification of positive and negative effects into processes and outcomes. Elements from the Theoretical Domains Framework [94] were drawn from to classify effects of CWAs on behavior. In order to produce a comprehensive taxonomy for all described positive and negative effects of CWAs in the health care field, we added new items to our taxonomy whenever any unique item was found in a paper. Whenever these items came from a specific theoretical framework, we noted the name of the framework and attempted to label the item using the same terminology as the original source framework.

##### Barriers/Facilitators

A second thematic content analysis was performed on the data regarding barriers and facilitators to the use of CWAs in health care with the initial coding scheme reflecting an existing framework concerning the determinants of ICT adoption [78]. Many new determinants of social media were inductively added to this framework. Our 3 reviewers created new codes for units of text that could not otherwise be coded using the original framework, thus refining and expanding the list. We also systematically searched each article to determine if a theoretical framework was used to report barriers and facilitators. If so, relevant elements were also added to the existing framework.

### Stage 6: Consulting Knowledge Users

As specified in our published protocol [86], we held meetings with representatives from the organizations involved (ie, the Association of Faculties of Medicine of Canada (AFMC), the International Medical Informatics Association (IMIA), the Federation of Patients and Consumer Organization in the Netherlands (NPCF), and the Pan American Health Organization (PAHO)) at the beginning, midway, and draft manuscript stages of this research in order to generate results that were useful for these knowledge users. Knowledge users were selected to represent a broad range of potential stakeholders representing medical education (AFMC), public health (IMIA and PAHO), and patient representatives (NPCF).

## Results

### Stages 1, 2, and 3: Mapping of the Literature and Study Selection

**Figure 1:**
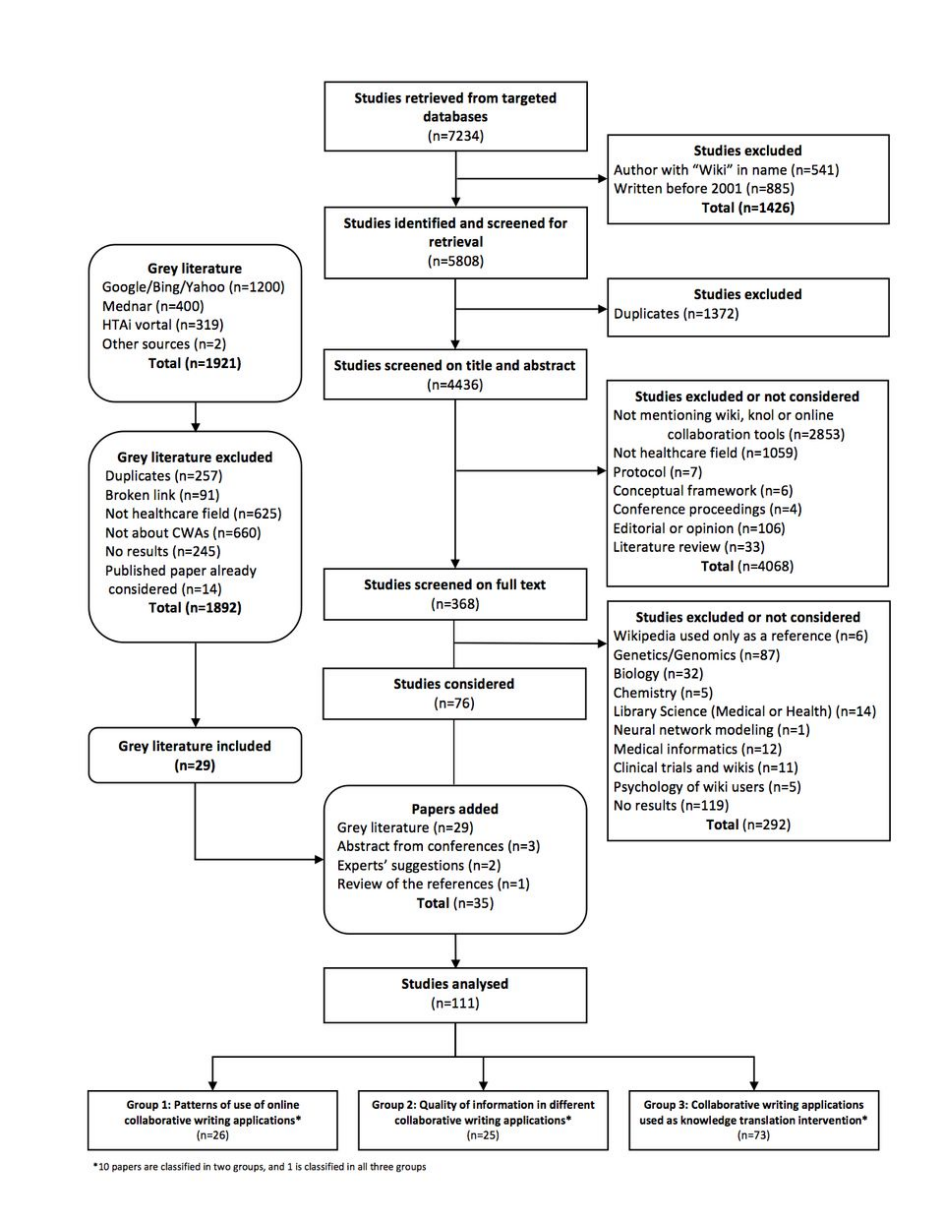
Flowchart of our mapping process and study selection.

After removing duplicates (n=1372), we screened the title and abstract of 4436 citations as well as the studies/abstracts from the grey literature, conference proceedings, expert consultation, and reviewing of reference lists (Figure 1) . All disagreements (n=794) were resolved through discussion.

**Figure 2:**
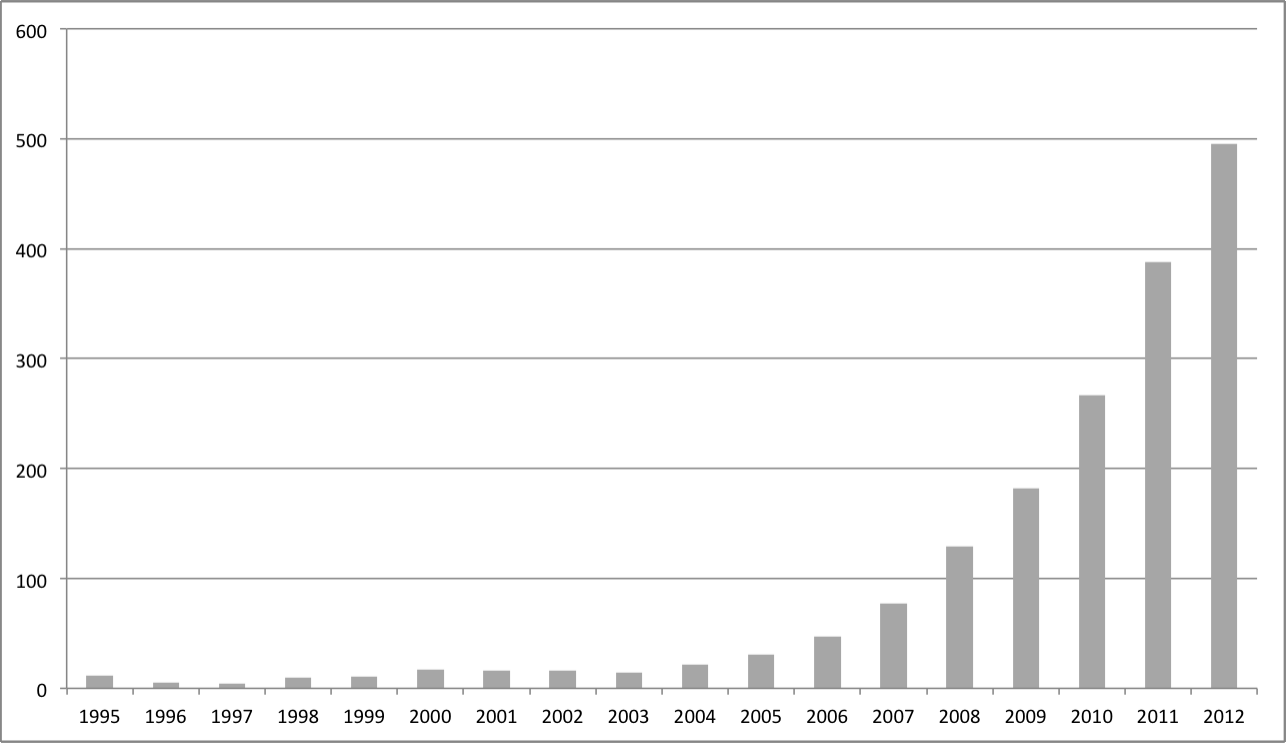
Histogram of the number of publications related to our search strategy per year.

Crowdsourcing identified two studies through Google Docs that were excluded. After review, we included 111 citations. Among these 111 citations, there were 28 abstracts without published full text but with sufficient results to be included. Twenty-six studies were grouped into Theme 1 (use patterns of CWAs), 25 into Theme 2 (quality of information in different CWAs), and 73 into Theme 3 (use of CWAs as a knowledge translation intervention). Figure 2 shows the rapid growth of the number of publications for the period within our search strategy.

### Stages 4 and 5: Charting Data, Collating, Summarizing, and Reporting Results

#### Study Characteristics

**Figure 3:**
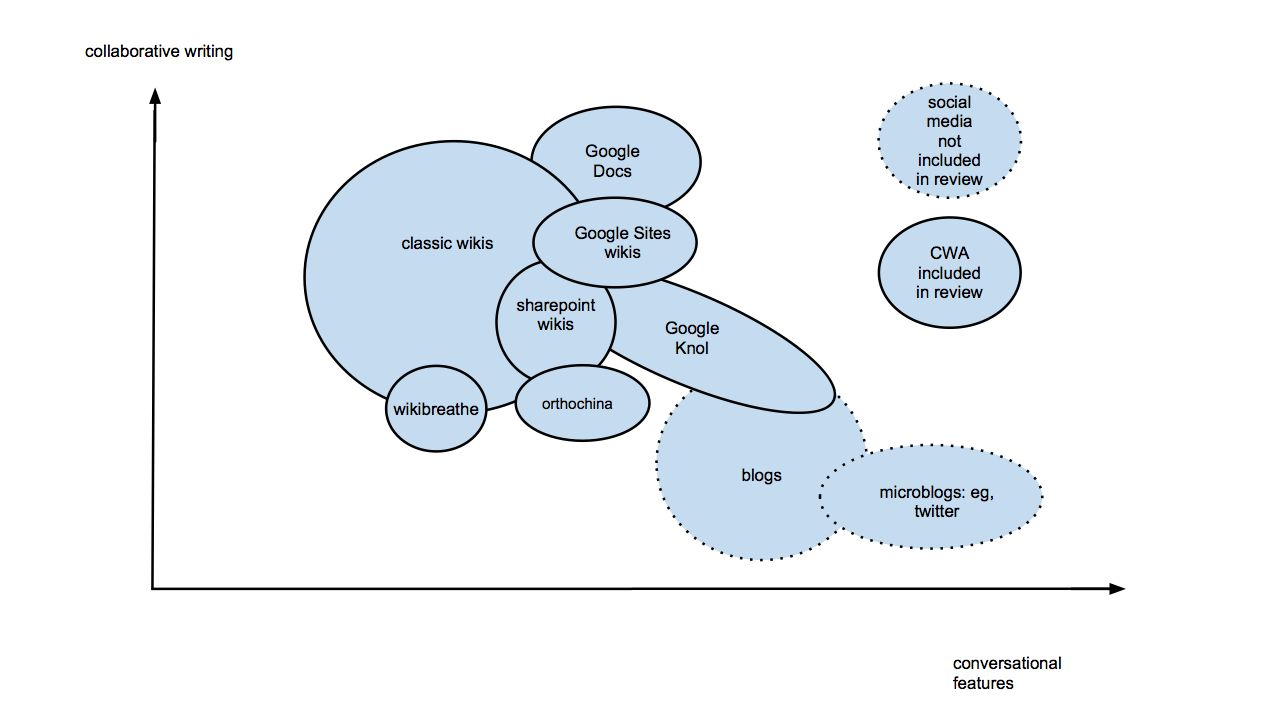
Collaborative writing applications Venn diagram.

We found 4 experimental studies, 5 quasi-experimental, 5 observational analytic, 52 case studies, 22 describing the quality of wikis, and 23 surveys on wiki use (Multimedia Appendix 1; [27,29-32,38,42,53,54,58,61,63,72,74,76,95-262]). Wikis (n=106) and Google Docs (n=6) are the main types of CWAs used in health care. One grey literature report compared Google Knol to Wikipedia [96]. Wikipedia was the focus of a large number of studies (n=36). The most frequently used wiki software were MediaWiki (n=44), PBworks (n=8), Wikispaces (n=6), Wetpaint (n=6), Microsoft SharePoint (n=3), and Google Sites (n=3). One paper described two wikis using Semantic MediaWiki (WikiEcho [97] and WikiDoc [98,99]). There were studies describing custom-built hybrid wikis (Wikibreathe (n=2) [100,101], Orthochina (n=1) [102], and FreyaWIKI (n=1) [103]; the use of virtual learning environments (eg, Blackboard) to host wikis as aids for supporting educational activities (n=8); and the use of more sophisticated social media platforms (eg, Drupal [104], MijnZorgNet [105], Atlassian [76], and MinJournal [106]) that offer wikis and other social media such as blogs and social networking services. The importance of the collaborative writing features compared to conversational features for each of the CWA studied are presented in a Venn diagram (Figure 3). This diagram shows that wikis and other hybrid wikis are centered more on their collaborative writing features compared to Google Knol, whose conversational features stand out more. Google Docs is different in that it offers both collaborative writing features (eg, real-time online editing) and conversational features (eg, linking documents to authors’ email allowing them to discuss a document while it is being created).

Two of the six studies pertaining to Google Docs were experimental [27,107]. The two other experimental studies were conducted with wikis [108,109]. As seen in Multimedia Appendix 1, the types of reported outcomes varied greatly depending on the context, goal, and framework used. Most outcomes concerned intermediate self-reported outcomes (eg, self-efficacy, usability scores, user satisfaction, dialogical communication scores), and some observed process outcomes (eg, wiki usage and contribution statistics, pre/post-test knowledge scores, quality of information, readability scores, number of communications). One study measured patient-oriented outcomes, such as blood pressure, physical activity, and cholesterol levels [107].

#### Use Patterns of CWAs

**Figure 4:**
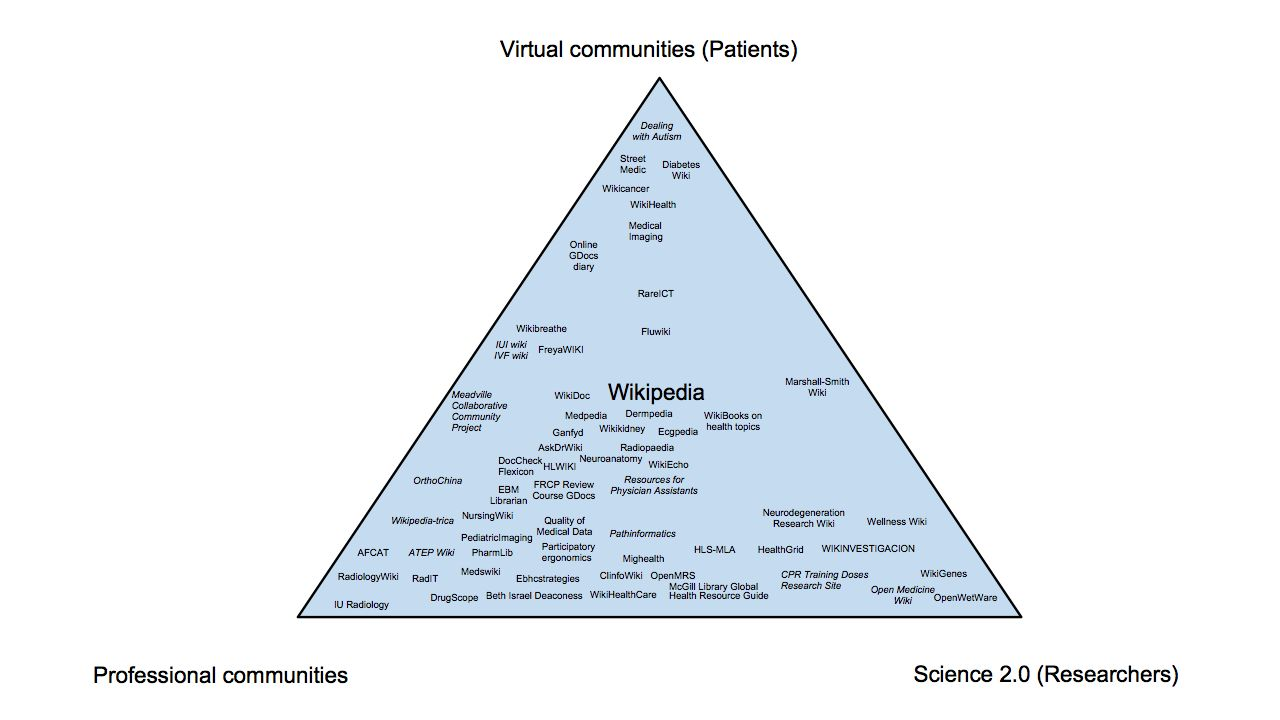
Medicine 2.0 map of the different collaborative writing applications (CWAs) and their users described in the included studies.

We found a total of 26 studies that presented different patterns of CWA use in health care: who uses the different CWAs, how much, and for what reasons (Multimedia Appendix 2; [29,42,53,110-130,189,263]). Most of these studies were conducted in the United States, United Kingdom, or Australia, and 1 and 3 studies were performed in Spain and in Canada respectively. All studies were published after 2006. Study populations varied widely including health care professionals (n=12), students (n=9), consumers (n=4), teachers/educators (n=2), scholars (n=1), and librarians (n=1). Most recurrent reasons for use were for academic purposes (case-based learning, e-learning, use of Web 2.0 tools for teaching) [110-115,264], for clinical purposes (to support patient care, to obtain drug information, to stay updated) [53,111,116-118], for personal use (by health care professionals and students) [42,118-121] and for seeking health information [122-127] or about specific diseases [128,129]. Other reasons were to update a scoping review [130] and to seek multiple stakeholder input [100,105]. Figure 4 shows that most CWAs described involve peer-to-peer communication between health professionals, followed by CWAs used by patients and researchers respectively.

In general, CWA use varied depending on the training level (eg, 70% or 132/188 first-year medical students using Wikipedia vs 37% or 86/234 third-year medical students [124]), the field of practice (eg, 9% or 4/44 pediatric neurologists used wikis [120] vs 35% or 369/1056 pharmacists [116]), and reason for use (eg, 100% or 51/51 radiology residents using a radiology department wiki [53] vs 15% or 360/2400 first-year psychology students using Wikipedia for personal information needs [121]). We found that a high prevalence of CWA use (ie, more than 50%) was reported in 58% (7/12) of surveys conducted with health care professionals and students (see Multimedia Appendix 2). The only longitudinal study conducted between 2005 and 2009 observed an increase in prevalence of Wikipedia use from 2% to 16% among undergraduate medical and biomedical students [123]. Another study reported higher use among younger medical students (480/593, 81%) compared to older consultants (215/389, 55%) [114]. Studies on the use of Wikipedia by pharmacists report rates of use ranging between 35% using this site for work-related questions in 2009 [116] to 72% using it mainly for personal reasons in 2011 [119]. For consumers, Wikipedia was ranked first when using search engines to find information about rare diseases [125] and to find information on generic drugs [126]. Wikipedia ranked as the second most consulted website both by a group of patients with Crohn’s disease [128] as well as by students searching for biomedical information [124]. While CWA rates of use are high, most reports present low rates of contributions to CWAs. From 6%-18% of students contribute to CWAs [114,115,121] while 3%-22% of junior physicians were reported to contribute to a CWA [42,264]. Furthermore, less than 1% of scholars were reported to contribute to a wiki project aiming at updating a scoping review [130]. Rarely, high rates of contribution were found in specific wiki projects [53,100].

#### Quality of the Information in Collaborative Writing Applications

We found 25 papers reporting on the quality of information in CWAs (Multimedia Appendix 3; [54,58,61,63,96,99,104,121,122,124,131-137,182,183,190,195-199]). With the exception of one paper evaluating the quality of information in 52 medical wikis other than Wikipedia [99], all studies focused on evaluating the quality of medical information in Wikipedia (n=24). No studies evaluated the quality of information within projects using Google Docs; however, one did compare the quality of information within Wikipedia and Google Knol [96]. Most studies (64%, 16/25) evaluated information destined to consumers while 32% (8/25) addressed the quality of information for students. Overall, 44% (11/25) of authors concluded that information within wikis and Wikipedia is partially reliable (ie, quality of information needs to be improved or updated) while 28% (7/25) reported that information within wikis and Wikipedia is not reliable and should not be used. Three studies reported no formal conclusion about quality of information [96,121,131]. Three authors concluded that medical information in wikis and Wikipedia was reliable and of high quality [54,104,132], yet only three used a validated quality assessment instrument [99,104,133]. Of the latter, one concluded that expert-moderated wikis could produce higher quality of information [99]. For example, wikis like WikiDoc [98], ECGpedia [234] and WikiKidney [230] were among the top-rated wikis in this study [99]. However, this study also concluded that all the wikis evaluated still needed improvements mainly concerning their completeness before they could safely be used for decision making. Another study concluded that Wikipedia was adequate for clinician and student education [104] while the third study concluded that further improvement of orthognatic surgery information was needed in Wikipedia before referring consumers to the site to support decision making [133]. A recurrent finding about Wikipedia was that its content is accurate, but that it often omits important medical facts and information [58,61].

As an educational tool, Wikipedia was reported to be comprehensive, of high quality, current, and appropriate for learning in gastroenterology and pathology [54,134]. However, variability in the content, accuracy, completeness, and referencing of drug information was reported [135]. Moreover, one study reported that 171 out of 271 (63%) of students do not verify the validity of references in Wikipedia articles [112]. While some think that Wikipedia should not be used by students as a source for referencing [135] or that it is unsuitable as a base for learning [63], others believe that its use by students need not necessarily be discouraged [136] and that it could be an informative and accurate source for education if used in combination with other learning materials [137]. Furthermore, one author considered CWAs to be excellent sources for continuing education and that they could represent the future of medical education as they allow for self-directed and supplementary education as well as for remote access [104].

#### Online Collaborative Writing Applications as Interventions

We identified four experimental studies in support of CWA use as educational and knowledge translation interventions (Multimedia Appendix 4; [27,107-109]). Three of these studies were conducted in the field of health professions education [27,108,109], and one was in the field of secondary prevention of cardiovascular disease in patients with previous acute coronary syndrome [107]. These studies found that the use of CWAs improved (1) physical activity and blood pressure control, (2) scientific writing skills among health science students, (3) medical student self-confidence and communication skills, and (4) nursing leadership skills. One study found that CWA use worsened diagnostic skills [108].

##### Taxonomy for Perceived Positive and Negative Effects Associated With CWAs

We classified the perceived positive and negative effects associated with CWAs into a taxonomy, covering eight categories (Table 2; [2,27,32,53,72,76,94,100,102,103, 105,107-110,122,130,138-178,265,266]).

In total, 57 positive effects and 23 negative effects were identified. Among the categories of positive effects that we found, the most frequently reported were that CWAs improve collaboration (n=41), positively impact learning (n=30), influence psychological domains (n=28), facilitate knowledge management and accessibility to information (n=30), improve efficiency of health care (n=19), improve quality of health care (n=6), and prevent disease (n=3). Among these effects, the Theoretical Domains Framework [94] was used to label and classify 22 of them into 3 psychological domains (self-efficacy, motivation, emotion) and 2 learning effects (skills and knowledge).

We found 2 studies referring to theoretical frameworks to describe their effects. Among the frameworks, the concept of communities of practice [266] was used to classify 3 studies reporting that CWAs improved the communication of tacit knowledge. The Dialogic Theory of Public Relations [265] was used to describe 5 positive effects wikis could have on public relations between health care organizations and consumers.

The most frequently cited negative effects were that CWAs could have unfavorable impacts on knowledge management (n=14) such as information overload (n=4) and fast dissemination of poorly validated information (n=4), as well as on certain psychological domains (n=6) such as added stress (n=1) and negative emotions (n=5). Some authors stated that CWAs could impede certain aspects of collaborative work (n=4) such as enhancing the perception of unequal work distribution (n=2) and encouraging conversation more than collaborative writing (n=1). Potentially serious negative effects of deletion of important medication information on Wikipedia by pharmaceutical companies (n=1) [177] and breaching of patient confidentiality (n=1) [179] were reported only in the grey literature.

##### Taxonomy for Barriers and Facilitators to the Use of CWAs in Health Care

A total of 48 barriers and 91 facilitators to the use of CWAs in health care were identified, of which 20 barriers and 69 facilitators were new determinants (Table 3; [32,53,54,76,100-102,106,109,110,114,116,130,141-143,145-149,153-156, 159,162-164,166-174,176,178,180,181,267-271]).

Among the latter, some were specific to social media (eg, social aspects of ICT, presence of a moderator, presence of a community of practice) and others were not (eg, information overload, mobile access, lack of proficiency in English). Although we found only 5 studies [101,109,153,155,156] that used a theoretical framework to identify barriers and facilitators, many of these barriers (n=11) and facilitators (n=34) were among those deemed as new.

The five barriers most frequently mentioned, in order of frequency, were unfamiliarity with ICTs (n=8), time constraints and workload (n=6), lack of self-efficacy (belief in one’s competence to use ICT) (n=6), material resources—access to ICT (n=5), worries about the scientific quality of the information (n=5), and the presence of a closed wiki protected by a password (n=5). The five most recurrent facilitators were having had training (n=12), scientific quality of the information (n=10), ease of use (n=8), triability (n=7), presence of a community of practice or a community of learners (n=7), and presence of a moderator (n=7).

**Table 2:** Positive and negative impacts of collaborative writing applications.

Impacts				Number of papers in which the impacts perceived as positive	Number of papers in which the impacts perceived as negative
**Processes (intermediate outcomes)** ^a^					
	**1. Effects on psychological domains** ^b^			28	6
		**1.1 Beliefs about capabilities (Self-efficacy)** ^b^			
			1.1.1 Self-Efficacy/empowerment: Not further specified	10 [32,108,163,122,138-143]	
			1.1.2 Empowering environment	2 [109,139]	
			1.1.3 Empowerment of families/relatives	1 [144]	
			1.1.4 Patient participation	3 [103,105,110]	
		**1.2 Motivation** ^b^			
			1.2.1 Engagement	7 [100,145-150]	
		**1.3 Emotion** ^b^			
			1.3.1 Satisfaction	5 [27,141,145,151,152]	1 [150]
			1.3.2 Loss of autonomy/feeling of being monitored		1 [32]
			1.3.3 Feeling of working in isolation		1 [153]
			1.3.4 Feeling of guilt about not participating		1 [109]
			1.3.5 Frustration due to technical issues		1 [154]
			1.3.6 Added stress		1 [155]
	**2. Learning effects**			30	1
		2.1 Subjective learning improvements: Not further specified		9 [108,140,141,114,145,150,152,156,157]	
		**2.2 Skills** ^b^			
			2.2.1 Communication skills eg, feedback	2 [138,151]	
			2.2.2 Handle fears and feelings	1 [158]	
			2.2.3 Adapt to different learning styles	4 [72,109,141,142]	
			2.2.4 Information and communication technology skills	1 [154]	
			2.2.5 Transfer of knowledge into practice	1 [138]	
			2.2.6 More efficient critiquing and evaluating the medical literature	1 [138]	
			2.2.7 Development of professionalism on students	1 [32]	
			2.2.8 Enhanced understanding of concepts	1 [159]	
			2.2.9 Decreased learning of diagnostic skills		1 [108]
		**2.3 Knowledge** ^b^			
			2.3.1 Knowledge (not further specified)	4 [72,109,154,160]	
			2.3.2 Awareness of guidelines	1 [161]	
		2.4 Better supervision by teachers		2 [141,154]	
		2.5 Better exam preparation		2 [108,110]	
	**3. Communication**			24	2
		3.1 Communication: Not further specified (impedes/improves)		9 [27,32,76,108,148,153,162-164]	2 [109,141]
		3.2 Feedback		2 [151,165]	
		3.3 Collegiality		1 [159]	
		3.4 Patient/health professionals communication		2 [144,146]	
		3.5 Communication of tacit knowledge^b^		3 [76,163,164]	
		3.6 Creates a network for families		1 [144]	
		3.7 Apomediation (communication process whereby individuals “stand by” to guide consumers to high quality information without being a prerequisite to obtain that information in the first place)^b^		1 [164]	
		**3.8 Dialogical communication between organizations and individuals** ^b^			
			3.8.1 Mutuality (the recognition of organization–public relationships)^b^	1 [122]	
			3.8.2 Propinquity (the temporality and spontaneity of interactions with publics)^b^	1 [122]	
			3.8.3 Empathy (the supportiveness and confirmation of public goals and interests)^b^	1 [122]	
			3.8.4 Risk (the willingness to interact with individuals and publics on their own terms)^b^	1 [122]	
			3.8.5 Commitment (the extent to which an organization gives itself over to dialogue, interpretation, and understanding in its interactions with publics)^b^	1 [122]	
	**4. Collaboration**			41	4
		4.1 Collaboration: Not further specified (impedes/improves)		23 [72,76,100,102,110,138-143,145-148,151,154,161,162,166-169]	1 [141]
		4.2 Reduces geographical barriers		11 [76,100,138,144,153,154,160,162,163,166,170]	
		4.3 Perceived unequal/equal separation of work		3 [100,110,141]	2 [141,154]
		4.4 Asynchronous communication		1 [163]	
		4.5 Wiki used as a conversational manner without contributing to the same text			1 [141]
		4.6 Define team responsibilities		1 [156]	
		4.7 Interprofessional collaboration		1 [105]	
		4.8 Creation of online presence		1 [156]	
	**5. Knowledge management and accessibility to information**			30	14
		5.1 Dissemination of information		8 [110,163,164,167,169,171-173]	
		5.2 Fast dissemination of poorly validated information			4 [102,159,164,174]
		5.3 Better access to information		8 [138,140,152,163,169,171,175,176]	
		5.4 Better exposure to world		1 [168]	
		5.5 Better knowledge translation across organizations		2 [146,164]	
		5.6 Centralized knowledge management		5 [140,152,156,164,166]	1 [110]
		5.7 Constantly updated information		1 [169]	
		5.8 Facilitates management of various content		1 [172]	
		5.9 Privacy issues health related data			1 [146]
		5.10 Spam/vandalism			2 [130,177]
		5.11 Updating of knowledge synthesis			1 [130]
		5.12 Saves paper		1 [175]	
		5.13 Information overload			4 [109,164,175,176]
		5.14 Wiki allows daily surveillance (looking for spurious edits)		1 [53]	
		5.15 Compiling anonymous data		1 [144]	
		5.16 Creativity/new ideas		1 [110]	
		5.17 Editing wars			1 [167]
**Outcomes**					
	**6. Efficiency of health care**			19	4
		6.1 Efficiency: Not further specified		5 [72,110,146,151,166]	2 [141,164]
		6.2 Saves money		1 [166]	
		6.3 Saves time/loses time		11 [32,102,146,148,152,155,161,163,166,169,170]	1 [162]
		6.4 Decreases/increases duplicate work		1 [164]	1 [155]
		6.5 Reduces workload		1 [174]	
	**7. Quality improvements**			6	2
		7.1 Quality improvements: Not further specified		5 [27,144,146,151,166]	1 [164]
		7.2 Wiki content didn’t meet users’ needs			1 [178]
		7.3 Reduces errors		1 [155]	
	**8. Disease prevention**			3 [107,142,146]	

^a^The Donabedian framework [93] for quality improvement was used to describe processes and outcomes.

^b^These items are processes that were taken from other psychological and organizational frameworks for change and used to describe and classify the effects of CWAs found in this review [2,94,265,266].

**Table 3:** Barriers and facilitators related to the use of collaborative writing applications.

Factors (Gagnon et al 2012 taxonomy)						Number of papers in which the factor was mentioned as a facilitator	Number of papers in which the factor was mentioned as a barrier
**1. Factors related to ICT (CWA)**							
	**1.1 Design and technical concerns**					13	8
		1.1.1 Readability of the information^a^					1 [171]
		1.1.2 Appearance of wiki (font, etc.)^b^				1 [101]	1 [159]
		1.1.3 Organization of information^b^				5 [101,163,169,171,180]	
		1.1.4 Immediately available technical information^a^				1 [166]	
		1.1.5 Having a sense of continuity and stability^b^[267]				1 [109]	
		1.1.6 References not intrusive in lay language texts^a^				1 [167]	
		1.1.7 Information overload^a^					2 [109,170]
		1.1.8 Mobile access^b^				1 [155]	
		1.1.9 Spam filter^a^				1 [130]	
		1.1.10 System can improve^a^				1 [154]	
		1.1.11 Rapid information changes^b^				1 [155]	1 [130]
		1.1.12 Design and technical concern – other					3 [109,142,154]
	**1.2 Characteristics of the innovation**					33	5
		**1.2.1 Ease of use/complexity**					
			1.2.1.1 Ease of content editing^a^			6 [106,163,166,170,176,180]	
			**1.2.1.2 Human/computer interactions** ^b^				
					1.2.1.2.1 Consistency (principle of minimum amazement)^b^[268]	1 [109]	
					1.2.1.2.2 Prevent error messages^b^[268]	1 [109]	
					1.2.1.2.3 Temporal contiguity (easy mental associations are made between verbal and visual)^b^	1 [109]	
			1.2.1.3 Reduce short-term memory load^b^[268]			1 [109]	
			1.2.1.4 Ease of use/complexity – other			8 [100,109,110,141,146,147,164,166],	4 [109,141,153,172]
		**1.2.2 Triability**					
			1.2.2.1 Permit Easy Reversal of Actions^b^[268]			3 [106,109,169]	
			1.2.2.2 Triability – other			7 [32,102,109,153,154,156,172]	
		1.2.3 Relative advantage (usefulness) or lack of					1 [130]
	1.3 System reliability					2 [109,169]	
	1.4 Interoperability (including Web browser interoperability)					3 [53,146,169]	2 [154,178]
	**1.5 Legal issues**					2	6
		1.5.1 Confidentiality - privacy concerns				2 [153,163]	3 [32,109,170]
		1.5.2 Liability^a^					1 [172]
		1.5.3 Copyright concerns^a^					2 [170,172]
	**1.6 Validity of the resources**					16	9
		1.6.1 Scientific quality of the information resources				10 [32,102,142,153,155,159,163,169,170,174]	5 [114,130,171,172,176]
		1.6.2 Content available (completeness)				2 [169,174]	2 [54,178]
		1.6.3 Appropriate for the users (relevance)				2 [53,176]	1 [178]
		1.6.4 Content updated frequently^a^					1 [54]
		1.6.5 Highly prevalent disease^a^				1 [130]	
		1.6.6 Rapidly growing body of research^a^				1 [130]	
	1.7 Cost issues: low human and hardware costs					3 [53,146,169]	2 [146,166]
	**1.8 Social aspects of ICT** ^a^					28	7
		1.8.1 Integrated support tools within wiki (toolbox, FAQ, forum, policies)^b^				6 [149,153,163,164,167,169]	
		1.8.2 Open access wiki^b^				1 [53]	5 [109,155,163,169,173]
		1.8.3 Good balance between restricted areas within wiki (private info) vs open areas (info for all)^a^				2 [106,130]	
		1.8.4 Interface linking content to conversations^b^				2 [109,180]	
		1.8.5 Use of template and seed with core set of pages^a^				4 [163,164,167,169]	
		1.8.6 Webmetric tool integrated with ICT to measure use (eg, Google Analytics) and contributions/authorship (eg, Wikigenes)^a^				1 [130]	
		1.8.7 Simultaneous real-time collaborative editing^a^				1 [109]	
		1.8.8 Gives informative feedback^b^[268]				1 [109]	
		1.8.9 Authorship transparent to increase reliability^a^				3 [130,169,174]	
		1.8.10 Socialization tactics (eg, welcome message)^a^				1 [130]	
		1.8.11 Controversial content^a^				1 [130]	
		1.8.12 Important impact on a large number of health professionals^a^				1 [130]	
		1.8.13 Lack of interest in topic^a^					1 [130]
		1.8.14 Wiki enabled with an RSS feed or email notifications (reminders)^b^				4 [32,109,159,163]	
		1.8.15 Inappropriate automatic computer editing^a^					1 [154]
**2. Individual factors or health care professionals characteristics (knowledge and attitude)**							
	**2.1 Knowledge**					1	12
		2.1.1 Awareness of the existence and/or objectives of the ICT					2 [130,141]
		**2.1.2 Familiarity with ICT**					
			2.1.2.1 Skills^b^[269]			1 [109]	
			2.1.2.2 Familiarity with ICT – other				8 [109,114,116,130,148,153,168,181]
		2.1.3 Lack of proficiency in English (the language of the Web)^a^					1 [146]
		2.1.4 Lack of knowledge about systematic review methods^a^					1 [130]
	**2.2 Attitude**					17	18
		**2.2.1 Agreement with the particular ICT**					
			2.2.1.1 Challenge to autonomy				1 [32]
			2.2.1.2 Outcome expectancy (use of the ICT leads to desired outcome)			1 [130]	
			2.2.1.3 Motivation to use the ICT (readiness)/resistance to use the ICT				4 [109,140,147,149]
			**2.2.1.4 Motivation to contribute to the wiki (desire to participate and post messages/information)** ^b^ **[269]**			3 [109,156,174]	1 [130]
					2.2.1.4.1 Motivation to contribute needs to be consistent with the person’s goals, plans, values, beliefs and interests^b^[269]	2 [109,156]	1 [130]
			2.2.1.5 Self-efficacy (believes in one’s competence to use the ICT)			6 [109,130,141,145,153,168]	6 [32,114,142,153,170,178]
			2.2.1.6 Preference for private learning environment compared to open environment^a^			2 [32,162]	
			2.2.1.7 Impact on personal life^b^[267]			1 [109]	
			2.2.1.8 Confidence in ICT developer				1 [116]
			2.2.1.9 Agreement with the particular ICT – other			1 [178]	2 [156,170]
		2.2.2 Agreement with ICTs in general (welcoming/resistant)				1 [174]	2 [114,168]
**3. Human environment**							
	**3.1 Factors associated with patients**					3	0
		**3.1.1 Patient/health professionals interaction**					
			3.1.1.1 Sharing of information between doctors and patients^a^			1 [174]	
			3.1.1.2 Sharing of information between doctors^a^			1 [174]	
			3.1.1.3 Sharing of information between patients^a^			1 [174]	
	**3.2 Factors associated with peers**					25	7
		**3.2.1 Support and promotion of ICT by colleagues**					
			3.2.1.1 Support by nurses^b^			1 [155]	
			3.2.1.2 Support by physicians^b^			1 [155]	
			3.2.1.3 Support by trainees^b^			1 [155]	
			3.2.1.4 Support and promotion by colleagues (not further specified)			3 [109,153,171]	
		**3.2.2 Other factors associated with peers (relations between colleagues)**					
			3.2.2.1 Credential verification^a^				1 [102]
			3.2.2.2 Frustration about having someone else edit personal contribution^b^				3 [106,109,141]
			3.2.2.3 Reluctance to team work^b^				3 [141,154,156]
			3.2.2.4 Using constructivist theoretical framework to setup a wiki is helpful^b^[270]			3 [109,153,156]	
			**3.2.2.5 Presence of a community of practice/community of learners** ^b^				
					3.2.2.5.1 Critical mass of scholars^a^	1 [130]	
					3.2.2.5.2 Presence of a small group of motivated editors^a^	1 [130]	
					3.2.2.5.3 Presence of community of practice/community of learners (not further specified)^b^	7 [76,106,109,149,156,169,174]	
			3.2.2.6 Openness, trust and respect^b^			4 [106,109,130,163]	
			3.2.2.7 Need for reciprocity (questions answered)^b^			2 [109,156]	
			3.2.2.8 Create teams of two collaborators working on same wiki page^a^			1 [162]	
**4. Organizational environment**							
	**4.1 Internal environment**					69	27
		**4.1.1 Work (nature of work)**					
			**4.1.1.1 Time constraints and workload**				
					4.1.1.1.1 Ultra-rapid decision making environment^b^		1 [155]
					4.1.1.1.2 Time constraints and workload – other	1 [32]	6 [109,114,141,148,162,170]
		**4.1.2 Resources availability**					
			4.1.2.1 Resources available (additional)				1 [116]
			**4.1.2.2 Material resources (access to ICT)**				
					4.1.2.2.1 Lack of constant Internet connection/access^b^		2 [146,155]
					4.1.2.2.1.2 Material resources (access to ICT) – other	6 [106,109,141,153,166,180]	5 [114,146,153,154,178]
			4.1.2.3 Human resources (IT support)			4 [109,154,156,171]	1 [146]
			4.1.2.4 Having a single platform^a^			1 [162]	
		**4.1.3 Organizational factors**					
			**4.1.3.1 Training**				
					4.1.3.1.1 Face-to-face training^b^	6 [32,76,141,149,153,156]	
					4.1.3.1.2 Use smaller groups (n=15-20) for one on one feedback^b^	1 [109]	
					4.1.3.1.3 Educators must be aware of human-computer interactions^b^	1 [109]	
					4.1.3.1.4 Training medical educators in using Web 2.0 ICTs^a^	1 [114]	
					4.1.3.1.5 Need for active learning/constructivist learning^b^		1 [109]
					4.1.3.1.6 Training –other	12 [53,76,109,141,143,145,148,153,154,159,163,169]	1 [146]
			**4.1.3.2 Management (strategic plan to implementing applications)**				
					4.1.3.2.1 Start with pilot project (implementation strategy)^a^	1 [162]	
					4.1.3.2.2 Index with Google - use Google Adwords (implementation strategy^a^)	1 [167]	
					4.1.3.2.3 Monitoring of use with Web metrics^b^	3 [130,156,167]	
					4.1.3.2.4 Management – other		2 [109,141]
			4.1.3.3 Presence and use of “champions”			1 [54]	
			4.1.3.4 Participation of end-users in the design			1 [172]	
			**4.1.3.5 Communication (includes promotional activities)**				
					4.1.3.5.1 Work with computer science department to implement a plan to generate traffic to wiki^a^	1 [167]	
					4.1.3.5.2 Getting new staff to participate for new look^a^	1 [163]	
					4.1.3.5.3 Encourage writers to contribute using their own style^a^	1 [163]	
					4.1.3.5.4 Forcing students to edit wiki^a^	1 [130]	
					4.1.5.5.5 Participating in a community of wiki editors^a^	1 [130]	
					4.1.5.5.6 Communication – other	3 [130,154,167]	
			**4.1.3.6 Ongoing administrative/organizational support**				
					4.1.3.6.1 Interactive Web applications permitted and unblocked within the health care institution^b^	1 [109]	
					4.1.3.6.2 Administrative/ organizational support – other	3 [109,130,156]	1 [114]
			**4.1.3.7 Incentive structures**				
					4.1.3.7.1 Giving continuing medical education (CME) credit^a^	1 [130]	
					4.1.3.7.2 New set of scholarly impact metrics^a^	1 [130]	
					4.1.3.7.3 Major cultural barrier in academia against participating in social media^a^		1 [130]
					4.1.3.7.4 Incentive structures – other	5 [54,102,109,162,169]	2 [130,172]
			4.1.3.8 Presence of a moderator^b^			7 [53,102,109,153,156,167,172]	
			4.1.3.9 Presence of metacognitive participants and dialogical participants^b^[271]			2 [109,156]	
			4.1.3.10 Accept that not all will participate and that lurkers will always exists/frustration about the lurkers who don’t contribute^b^			1 [109]	3 [141,149,154]
	**4.2 External environment**					1	1
		4.2.1 Financing of ICT/financial support					1 [109]
		4.2.2 Coupling traditional publications with wiki contributions^a^				1 [130]	

^a^These new determinants did not exist in the Gagnon et al framework

^b^These new determinants were identified in papers using a theoretical framework.

## Discussion

### Principal Findings

We confirmed that CWAs are currently being used frequently in health care, by a variety of stakeholders including patients, professionals, and researchers, for a large diversity of purposes. Our complete portrait of the literature shows that wikis are by far the most commonly studied type of CWA and that most studies had observational designs. Each type of CWA has different collaborative writing and conversational features that must be considered by decision makers when making a choice about which CWA to use in different collaborative projects. Many positive effects are attributed to the use of CWA in health professions education and knowledge translation. Further systematic synthesis of experimental and quasi-experimental evidence is needed before any clear policy recommendations can be made about implementing these tools in current practice. Moreover, there is an array of potential negative effects and barriers that need to be addressed in future primary research projects.

### The Use of CWAs in Health Care

Despite the controversy surrounding the use of information in Wikipedia in clinical decision making [57,65], a high proportion of health professionals and students are already using Wikipedia and other CWAs, with use apparently increasing, especially among younger professionals. Although more research is needed to confirm this trend, these findings are consistent with an overall trend to increased use of social media among health professionals [79,272]. Our systematic mapping of the literature shows that wikis are the most frequently studied type of CWA. Furthermore, the use of Wikipedia by students and professionals represents the focus of many of our included studies. Google Docs studies come second, and we found only one study about Google Knol. This is not surprising since Wikipedia is the sixth most visited website worldwide and appears in top 10 results of search engines concerning health questions [125]. However, as readership of Wikipedia is rapidly changing, it is important to acknowledge that usage percentages depend not only on how you ask the question but also when you ask the question. Moreover, Google terminated the Knol project in 2011 despite interesting health projects using this platform including the PLOS Currents: Influenza project [273,274]. Besides the single publication we found about Google Knol comparing Knol to Wikipedia [96], there are no published accounts of Google’s reasons for closing and transferring Knol to the Annotum platform.

Based on the Medicine 2.0 map [2], we demonstrated that current CWAs in use are mainly oriented towards health students and professionals’ peer-to-peer interactions. In fact, use of CWAs is a major area of research in health education [275,276]. In particular, of the 4 experimental studies identified, 3 were education studies showing that CWAs positively influenced learning processes and almost half (n=48) of all the studies in this review concerned health professions education. Albeit less common, there are also studies about CWAs involving consumers and professionals to co-create decision-making tools [100,101,105,277]. These four projects seem relevant given that patient-centered care has become a central aspect of knowledge translation and experts have called for new ways of involving patients in the implementation of evidence [278]. Another remarkable finding is that even fewer CWAs involve consumers and researchers in sharing hard to find phenotype information about rare genetic and congenital diseases [106,144].

Researchers are starting to explore the use of CWAs, for example in updating a scoping review [130]. Another expert/researcher driven wiki is the OpenMRS electronic medical record implementation wiki, an example of wikis’ full potential for improving health in developing countries. Although the World Health Organization is exploring the use of a wiki to update the 11th International Classification of Disease [49], we did not find any published accounts on their experience, nor did we find any related to the discontinuation of Medpedia [37]. The reasons for ending this ambitious project involving important stakeholders would provide lessons for the future.

### CWAs Features and Implications for Health Care

After comparing how each CWA was used in different collaborative writing projects, we found that wikis and certain hybrid custom-built wikis have collaborative writing features that are more prominent compared to their conversational features. These collaborative writing features produce artefacts of synthesized knowledge that lend themselves more readily to daily use than those produced from conversational knowledge. For example, using a wiki to store and update care protocols readily applicable to the care of emergency department patients would be more useful in daily practice than reading the discussion page found in support of the wiki page itself. Conversely, Google Docs, certain knowledge management applications (eg, Google Sites, Microsoft Sharepoint) and other social media platforms (eg, MijnZorgNet, Atlassian Confluence, MinJournal) integrate additional features that favor conversation and deliberation between users. These additional conversational features produce discussions between users about the knowledge being shared and add to users’ understanding about the content found on the collaborative writing pages of these applications.

### Effects of CWA and Wiki Use in Health Care

Most evidence stemmed from case reports and observational studies demonstrating perceived positive effects of CWA use in health care on behavior change, education, communication, collaboration, knowledge management and access to knowledge, and better quality and efficiency of health care. These findings support claims that CWAs and wikis facilitate that online professional communities create, share, and synthesize knowledge; increase access to health information; and offer opportunity for public participation and citizenship [84,276,279]. Although less frequently reported, we also found a series of perceived negative effects (ie, information overload, fast dissemination of poorly validated information, loss of autonomy, feeling of working in isolation, increased stress, perceived unequal distribution of tasks within teams, biased editing, editing wars, and vandalism/wikispam) that could mask some of the positive effects of CWAs. Innovative developments such as semantic wikis [8,97,98,276,280] and bots [11,281] may decrease some of these negative effects. For example, to reduce the impression of information overload, certain authors are exploring semantic wikis to better organize and structure information based on a logical ontology [97,98]. Semantic wikis could help organize the knowledge being shared [8,276,280], potentially improve its meaningful use [282,283] and eventually allow its integration into intelligent Web-based decision-support tools [280]. Other authors are exploring the use of bots to decrease the risk of vandalism, biased editing, and spam [11,281]. A bot is a computer program that runs automatically and continuously within wikis and can conduct simple tasks like correcting spelling and syntax. Wikipedia contains many different bots that help ensure its quality [281]. More complex bots exist like the one in WikiPathways that surveys the content and identifies potential inconsistencies, redundancies, and incomplete data [11].

### Barriers and Facilitators to the Use of CWAs and Wikis in Health Care

The use of CWAs in health care faces barriers that limit their use that are similar to those experienced in other fields: unfamiliarity with ICT [284], time constraints and workload [275], lack of self-efficacy to use CWAs [275], access to CWAs [285], worries about the scientific quality of the information resources [276,281,286,287], readability of information [281], the presence of a closed wiki protected by a password [276,281] and legal concerns [276,286,287].

A recurrent finding about the information in Wikipedia was that it is in large part accurate, free, and easy to access. However, even though Wikipedia does not recommend including medication doses due to concerns about errors [288], it is often incomplete and can lack appropriate referencing of medical information [58,61], thereby possibly indirectly causing patient harm [135]. One observational study demonstrated that involving moderators and experts in the sharing and curation of information within CWAs improves the quality of information [99]. However, as previous authors have demonstrated, finding ways to get these experts to participate remains a challenge [4,130,182,276,289].

Maintaining high-quality information as well as high contribution levels is a heated debate with opposing views (ie, password-protected wiki vs open wiki) [53,105,109,155,163,169,173]. Authors from multiple fields have explored modalities to stimulate participation [276,281,284,285,290-296]. Many facilitators reported from fields other than health care include training [284,296], scientific quality of the information resources [281,286,287], ease of use [291], having access to integrated support tools [296], ease of content editing [297-299], access to CWA [285], self-efficacy [300,301], and the use of incentives [293,294,302-304]. Some propose a set of scholarly metrics that would reward contributions to collaborative projects [130]. The journal *RNA Biology *stimulates contributions to Wikipedia by scholars by requiring that manuscripts be summarized for a Wikipedia page before accepting to publish the article [305]. The WikiGenes project has recognized the importance of authorship [10,36]. Finally, similar to other fields [293,294,297,306], the presence of a community and the sense of community is a frequently reported facilitator that increases contributions by health care stakeholders. Experts suggest that studying CWAs involves looking at both the technology and its community of users [276,285,307]. Thus, understanding the success of a project using a CWA must also include exploring the fundamental elements of communities of practice [266]. Communities of practice can meet online (ie, virtual community) or face to face. Similar to systematic reviews on communities of practice [308,309], our scoping review identified the presence of a moderator and/or a champion as a key factor for a successful collaborative writing project. Related to the concept of community, the success of a collaborative writing project also includes having a critical mass of participants, shared values, openness, trust, and respect.

### Clinical Relevance

We believe that our findings are important for consumers, professionals, researchers, and health care organizations around the world that are already using CWAs and/or planning to use a CWA to improve health care. Although we have found some evidence from experimental studies to support the use of CWAs as a health profession’s educational intervention and a large body of observational evidence supporting the use of CWAs as a knowledge translation intervention, a formal systematic review should be conducted to further synthesize the evidence and conduct a formal risk of bias assessment before making practice recommendations. Furthermore, the implementation of CWAs is fraught with barriers and the potential for adverse effects, requiring primary research to assess their safety.

Unfortunately, the breadth and depth of the literature on the use of CWAs specific to public health is scarce. However, based on some ongoing and promising projects [49,76,99,139,146,164], it is clear that the uses of CWAs for public health are vast and far-reaching. Although more research is needed within this specific domain, CWAs improve information access, collaboration, and can improve health education—all tenets of public health. Patients and consumers often experience many barriers in the use of CWAs, with information quality being among the most reported. The readability of articles within Wikipedia is a key area that must be addressed, as it will improve health literacy and knowledge translation [310]. There are also promising projects that may shed light on the effectiveness of involving patients in the development of clinical of guidelines [311]. Evidence from experimental studies about engaging patients with CWAs is still rare and needs to be replicated in robust prospective trials before making recommendations.

### Strengths of This Study

This is the first study that has conducted a scoping review to examine the depth and breadth of evidence about the use of CWAs in health care. We rigorously followed scoping review methodology and conducted a systematic and broad search of CWA use in multiple scientific databases and grey literature sources. A scoping review was the ideal methodology to employ for a number of reasons. First, it is an explorative method used when the relevant literature is considered to be broad and diverse [312]. Moreover, the study of these applications is an emerging field that is being examined with diverse methods [28,32,61], with different theoretical frameworks [29] and in different contexts [46,313]. We used a high-quality collaborative Web-based software to manage our review, to import studies, to extract data and to create reports. Every step of our review has been extensively described. By including knowledge users and policy makers, we have produced a relevant synthesis of the evidence targeting their needs. Based on empirical results, this scoping review has also extended an existing taxonomy of adoption determinants to the study of a social media application. The original taxonomy had been developed using a rigorous mixed-methods systematic review methodology [78]. Although our new extended taxonomy is very comprehensive, we believe that this level of detail was important to maintain in order to help future researchers explore the impact of these barriers and facilitators. Moreover, we have also created a new taxonomy of effects based on elements from other sociocognitive and organizational frameworks of change. Our use of the Donebadian framework was very useful because of its generalizability and overarching broad scope. Other more specific frameworks (eg, Theoretical Domains Framework) fit well within this overarching framework. Research should validate our two new taxonomies for future development, assessment, and implementation of other social media applications.

### Limitations of This Study

Even though we did everything possible to minimize publication bias by systematically and extensively searching for any sources of the grey literature presenting negative results (eg, including a lay media newspaper article [177]), we believe publication bias is not excluded. For example, we have not found published reports explaining the failed attempts at maintaining Google Knol or Medpedia. Many other CWAs sites have also disappeared over the course of the years without any clear explanations. In 2009, David Rothman had listed 69 medical wikis, many of which are now inactive or simply do not exist anymore [39]. Such reports describing the reasons for CWA failure would help generate important lessons for the advance of the science of collaborative writing.

Second, our scoping review methodology [87,88] did not include formal quality assessment. However, we classified studies based on the strength of their design in order to help us identify areas for primary research and those that produced sufficiently robust evidence for making recommendations.

Third, our scoping review was limited to reviewing CWAs using a definition that excluded related applications like blogs, microblogs, discussion forums, and patient communities (eg, PatientsLikeMe). Even though these social media applications are collaborative as well and share some common features with CWAs, we believe that it is important to study them separately to better understand each application’s impact and interaction with other social media.

Finally, our search strategy is limited to studies published between January 1, 2001, and September 16, 2011, while several more recent studies about CWAs have been published [263,272,314-319].

### Unanswered Questions and Future Research

This scoping review has identified a number of research gaps. There is a need to conduct systematic reviews to further synthesize the results of experimental and quasi-experimental studies in the field of health professions education and to further synthesize evidence about implementation strategies addressing the different barriers identified. Given that the majority of the literature presently exists in the form of case reports with self-reported measurements, it is essential that further prospective trials with objective outcomes be conducted. Future trials should identify implementation processes that can be influenced by CWAs and how to measure them (possibly using Web metrics [130,167,276]) as intermediate outcomes of a complex knowledge translation intervention. In this respect, in addition to other frameworks defining evaluation plans of dynamic collaborative applications [320], our taxonomies of CWA adoption determinants and effects will help plan such trials. This will help researchers understand the different mechanisms of action at play leading to improved patient-oriented outcomes (quality of life, morbidity, mortality). Although the feasibility of conducting a randomized clinical trial to study the effectiveness of CWAs seems daunting, other complex interventions have been studied using this methodology [321].

Before conducting such trials, researchers and decision makers must reflect on defining the purpose of using a CWA as a knowledge translation intervention. Researchers must also find ways to adapt CWAs to the particular needs of different stakeholder groups (consumers, professionals, and researchers). Important barriers such as the quality of information contained in different wikis must be better addressed. As previous authors have stated [183,320], measuring the quality of user-generated content and its change over time is a challenging task requiring research [322]. Finding ways of assuring the scientific integrity of evidence within CWAs and recognizing authorship are significant stumbling blocks that need to be addressed for health care [102,114,130,171,176,323]. Studying each specific behavior involved in using CWAs (ie, to use, to contribute, to edit, to delete) with the help of theoretical frameworks will also help inform future interventions.

In addition to other technical considerations [324,325], future studies should explore the impact of collaborative writing and conversational features on information sharing and investigate what kind of knowledge (explicit vs tacit [266]) is shared. This could help knowledge users choose an appropriate CWA. As future communication tools, the impact of using different types of media embedded within CWAs (audio and video recordings) should also be explored. Finally, an important consideration to explore in future studies would be to determine the impact of using a closed vs an open CWA on the quality of the information found within the CWA and on the type of barriers experienced by users.

### Conclusion

The prevalence of CWA use is high in various fields of health care, and they are used for a variety of purposes. They present many potential positive and negative effects as knowledge translation tools. Although we found some experimental and quasi-experimental evidence in favor of using CWAs as educational and knowledge translation interventions, the vast majority of included studies were observational case reports about CWAs being used by health professionals and patients. More research is needed to determine which stakeholders benefit the most from using CWAs, to address the barriers to their use, to find ways to ensure the quality of their content, to foster contributions, and to make these tools effective knowledge translation tools for different stakeholders. Answers to these questions are needed before clear policy recommendations can be made about the safe use of CWAs in health care.

## Multimedia Appendix 1

Characteristics of included studies.

## Multimedia Appendix 2

Patterns of use of collaborative writing applications.

## Multimedia Appendix 3

Quality of information in collaborative writing projects.

## Multimedia Appendix 4

Characteristics and results of experimental studies.

## Abbreviations

AFMC: Association of Faculties of Medicine of Canada
CWA: collaborative writing application
ICT: information and communication technologies
IMIA: International Medical Informatics Association
NPCF: Federation of Patients and Consumer Organization in the Netherlands
PAHO: Pan American Health Organization
