# Supplementary material for: Wikis and Collaborative Writing Applications in Health Care: A Scoping Review
Source: J Med Internet Res. 2013 Oct 8;15(10):e210. doi: 10.2196/jmir.2787 (PMC3929050; doi:10.2196/jmir.2787)
Supplement: Supplementary file 1 [file jmir_v15i10e210_app1.pdf]

Theme	Authors, Year of publication, Type of publication*	Study design	Type of CWA (name of CWA and URL if available)	Software used	Context of study	Speciality	Type of outcomes reported
**1**	Alkhateeb (2011) [119]	Survey	Wiki (Wikipedia)	MediaWiki	Describe social media use among pharmacists in West Virginia	Pharmacy	Prevalence of use of different social media
**1**	Archambault (2010) (G, A) [29]	Case study	Google docs	Google Docs	Describe the use of a Google Docs slideshow	Emergency medicine	Frequency of use
**1, 3**	Bender (2011) (G) [130]	Case study	Wiki (*Open Medicine* wiki http://wikisr.openmedicine.ca) [261]	MediaWiki	Updating of a scoping review [261]	Asynchronous telehealth	Usage statistics and comments from users
**1, 3**	Brokowski (2009) [116]	Survey	Wiki (*Wikipedia*)	MediaWiki	Pharmacists using Wikipedia for medication information	Pharmacy	Prevalence of Wikipedia use and reason for use
**1**	Dodson (2011) [111]	Survey	Wiki	PmWiki (http://www.pmwiki.org) [185], b2evolution (http://b2evolution.net) [186] and Libguides (http://libguides.com) [187]	Survey of health sciences libraries usage of blogs and wikis	Medical education, healthcare library science	Departments using wikis; type of wiki and content management software used; purposes of using wikis; description of their use of wikis and best practices reported
**1**	Gonzalez de Dios (2011) [120]	Survey	Wiki and Google Docs	N/A	Survey of Web 2.0 resources used by clinicians in Spain	Paediatric neurology	Prevalence of use of different social media applications
**1, 3**	Gupta (2010) (A) [100]	Case study	Hybrid wiki (*Wikibreathe/OCTAPUS tool*: http://knowledgetranslation.ca/octapus/login.php)	Custom-built application***	Development of an asthma action plan	Respirology	System Usability Scale; wiki usage statistics; overall satisfaction
**1**	Harris (2010) [112]	Survey	Wiki (Wikipedia)	MediaWiki	To examine student	Mental health	Prevalence of use

					approaches to using Wikipedia as a reputable resource		
**1, 2, 3**	Hickerson (2009) (G) [122]	Survey	Wiki (*Wikipedia*; *WikiHealth* (www.wikihealth.com) [260])	MediaWiki****	Investigation of how wikis facilitate dialogue between consumers and healthcare organisations	Public relations	Measured levels of perceived dialogical communication scores
**1,3**	Hughes (2009) [42]	Survey	Wiki (*Wikipedia*)	MediaWiki	Junior physicians use of Web 2.0 resources	10 different specialties	Opinion of junior physicians about using wikis
**1**	Iyer (2011) (D) [117]	Survey	Wiki (*Wikipedia*)	MediaWiki	Drug information-seeking behaviours among health care professionals	Pharmacy	Wikipedia usage statistics
**1**	Judd (2010) [123]	Observational analytic design	Wiki (Wikipedia)	MediaWiki	Biomedical students' on-campus use of Internet	Healthcare Education	Most frequented web sites and technologies
**1, 2**	Judd (2011) [124]	Observational analytic design	Wiki (Wikipedia)	MediaWiki	Determine how undergraduate medical students used five popular sites to locate and access biomedical resources	Medical Education	Internet usage logs; students' perceptions of each site's usefulness and reliability
**1, 3**	Kohli (2011) [53]	Survey	Wiki (*IU Radiology:* www.indyradres.org*) [95]*	Dokuwiki (www.dokuwiki.org) [188]	Centralized knowledge management system to share useful documents for radiology residents (e.g., dictation	Radiology, residency training	Resident acceptance survey

					templates, phone numbers, etc.)		
**1**	Laurent (2009) [125]	Observational analytic design	Wiki (Wikipedia)	MediaWiki	Determine the significance of English Wikipedia as a source of online health information	General health information	Wikipedia's ranking on general Internet search engines; cumulative incidence and average position of Wikipedia compared to other Web sites among results on Internet search engines
**1**	Law (2011) [126]	Observational analytic design	Wiki (Wikipedia)	MediaWiki	To investigate the sources of online information about prescription drugs used by consumers	Pharmacy / toxicology	Number of times a Web site appeared as the first result in search engines; Wikipedia page hits for prescription drugs in 2008 and 2009
**1**	Lemley (2009) [113]	Survey	Wiki	N/A	To investigate which social networking tools are being used in the curricula of medical and nursing schools	Medical and nursing education	Prevalence of use
**1**	Limdi (2011) (A) [128]	Survey	Wiki (*Wikipedia*)	MediaWiki	Prospective study of patients attending Inflammatory bowel disease clinics	Gastroenterology	Ranking of popularity of different web sites and their trustworthiness
**1**	Martin (2011) (G, A) [127]	Survey	Wiki (Wikipedia)	MediaWiki	To assess the information literacy skills of first year pharmacy students	Pharmacy, education	Search strategies used by students
**1, 3**	Sandars (2007-a) [114]	Survey	Wiki	N/A	Survey of medical students and qualified medical practitioners	Medical Education	Survey to determine familiarity with different social media; barriers to their use
**1, 3**	Sandars (2007-b) [189]	Survey	Wiki	N/A	Survey of students skills, experience and	Medical Education	Previous use of wiki, attitudes towards wikis

					views on information technology		
**1**	Sandars (2008) [115]	Survey	Wiki (Wikipedia)	MediaWiki, PBworks	To identify the extent of use of social media by medical students	Medical Education	Prevalence of use
**1**	Santos Arrontes (2007) [129]	Survey	Wiki (Wikipedia)	MediaWiki	To evaluate the use of Internet by patients from a urology clinic	Urology	Ranking of the most visited web pages
**1, 2**	Schweitzer (2008) [121]	Survey	Wiki (Wikipedia)	MediaWiki	To examine Wikipedia's coverage of psychology-related concepts, its accessibility, and to describe how undergraduate students use Wikipedia	Mental health	Coverage; use of Wikipedia for personal use, school-related work, and as a formal reference in academic work
**1**	Usher (2011) [118]	Survey	Wiki	Twine: www.twine.com (now bought by www.evri.com)	Types of social media used by allied health professions in Australia	Healthcare in general	Prevalence of use
**1, 3**	Williams (2011) [110]	Survey	Wiki	Wikispaces	Student use of wikis to support problem-based learning	Emergency medical services, education	Student attitudes on group work using wikis
**2**	Aldairy (2011) [133]	Descriptive quality assessment of wiki content paper	Wiki (Wikipedia)	MediaWiki	Dentofacial deformities	Oral and maxillofacial surgery / orthodontics	Quality and reliability of UK websites providing information on orthognathic and jaw surgery to patients
**2**	Ayes (2010) (A) [190]	Descriptive quality assessment	Wiki (Wikipedia)	MediaWiki	Overdoses and poisoning / toxicologic	Toxicology	Comparison of Wikipedia toxicology content with content from Poisindex

		of wiki content paper			emergencies		
**2**	Clauson (2008) [61]	Descriptive quality assessment of wiki content paper	Wiki (Wikipedia)	MediaWiki	Quality of drug information in Wikipedia	Pharmacy	Scope, completeness, and accuracy of drug information in Wikipedia compared to that of a Medscape Drug Reference
**2**	Czarnecka-Kujawa (2008) (A, G) [134]	Descriptive quality assessment of wiki content paper	Wiki (Wikipedia)	MediaWiki	Study on the comprehensiveness, reliability and readability of Wikipedia concerning ICD-9 and ICD-10 gastroenterology diagnostic codes	Gastroenterology	Comprehensiveness, reliability and readability
**2**	Devgan (2007) (A, G) [58]	Descriptive quality assessment of wiki content paper	Wiki (Wikipedia)	MediaWiki	Internal validity of Wikipedia as a medical and surgical reference	Surgery	Quantitative metrics of quality (number of edits, unique editors, and references) and evidence of qualitative rigour (accuracy, completeness, discussion of indications and risks, and suitability for patients).
**2**	Dobrogowska-Schlebusch (2009) [99]	Descriptive quality assessment of wiki content paper	Wiki (see footnote for the list of names of wikis evaluated ***********)	See footnote to see the names of software used for each wiki**********	Assessment of the quality of 52 medical wikis using Health Summit Working Group Quality Criteria and experiences with another wiki created for migrant health issues in Europe (http://mighealth.net/eu/)	Public health	Credibility (source, currency, relevance/utility, editorial review process for the information); Content (accuracy, completeness, disclaimer); Disclosure (purpose of the site, private policy); Links (selection, architecture, content, back linking); Design (accessibility, navigability, internal search capability); Interactivity; Caveats (clarification of whether site function is to market products and services or is it a primary information content provider)
**2**	Friedlin (2010) [132]	Descriptive quality	Wiki (Wikipedia)	MediaWiki	Logical observation	Medical taxonomy	Number of articles in Wikipedia that are exact matches, partial matches

		assessment of wiki content			identifiers names and codes (LOINC) database covered in Wikipedia		or mismatches with parts of the LOINC database
**2**	Haigh (2011) [136]	Descriptive quality assessment of wiki content paper	Wiki (Wikipedia)	MediaWiki	Assessing the quality of Wikipedia article references and sources used by nursing students	Nursing, education	Mean number of reputable sources per Wikipedia entry
**2**	Hanson (2011) [104]	Descriptive quality assessment of wiki content paper	Wiki (Dermpedia: www.dermpedia.org [191]; Medpedia: http://web.archive.org/web/20130115090302/http://www.medpedia.com/about [37] and Wikipedia)	(Dermpedia: http://drupal.org; [193] Medpedia: MediaWiki; Wikipedia: MediaWiki)	Sampling of the top dermatology Internet resources, as assessed by a group of medical students	Dermatology	Websites ranked by using a matrix derived from the Silberg Criteria
**2**	Johnson (2008) [131]	Survey	Wiki (Wikipedia)	MediaWiki	Rare medical entities which physicians in training would not be expected to know	Primary care and internal medicine	Frequency of searching different databases; efficiency of different search engines (number of links to find answer); correctness of answer
**2, 3**	Kim (2010) [54]	Descriptive quality assessment of wiki content paper	Wiki (*Pathology informatics curriculum wiki: http://pathinformatics.wikispaces.com) [75]*	Wikispaces	Need for informatics training as part of pathology training	Pathology, medical informatics, education	Quality of Wikipedia pages linked to the Association for Pathology Informatics curriculum
**2**	Lavsa (2011) [135]	Descriptive quality assessment of wiki content paper	Wiki (Wikipedia)	MediaWiki	Assess the accuracy, completeness, and referencing of medication information in Wikipedia	Pharmacy	Accuracy, completeness, and referencing (fully, partially, or none)
**2**	Leithner (2010) [195]	Descriptive quality assessment	Wiki (Wikipedia)	MediaWiki	Scope, completeness, and accuracy of	Oncology	Scope, completeness, and accuracy of information

		of wiki content paper			information found on osteosarcoma in Wikipedia		
**2**	Lorenz (2010) [183]	Descriptive quality assessment of wiki content paper	Wiki (German-language Wikipedia)	MediaWiki	To assess the quality of articles on dentistry in Wikipedia	Dentistry	Number of scientific quality of articles
**2**	McInnes (2011) [182]	Descriptive quality assessment of wiki content paper	Wiki (Wikipedia)	MediaWiki	Readability of websites on various causes of disease	General health information	Gunning FOG, SMOG, Flesch-Kincaid and Flesch Reading Ease tests
**2**	Mercer (2007) [196]	Descriptive quality assessment of wiki content paper	Wiki (Wikipedia)	MediaWiki	Review the handling of mental health topics in Wikipedia	Mental health	Shortcomings of Wikipedia articles on autism and other mental illness
**2**	Mühlhauser (2008) [197]	Descriptive quality assessment of wiki content paper	Wiki (Wikipedia)	MediaWiki	Using evidence-based medicine criteria, Wikipedia was compared to two major German health insurances for content and presentation of patient information	General health information	Quality of information based on a checklist containing 11 evidence-based criteria groups
**2**	Pender (2009) [63]	Descriptive quality assessment of wiki content paper	Wiki (Wikipedia)	MediaWiki	Compare the quality of entries on multiple sclerosis, otitis, conjunctivitis in Wikipedia and three traditional non-wiki databases	Medical Education	Accuracy, coverage, concision, currency, suitability, accessibility and useability of the resources

					(UpToDate, eMedicine, AccessMedicine)		
**2**	Rajagopalan (2010) [198]	Descriptive quality assessment of wiki content paper	Wiki (Wikipedia)	MediaWiki	Compare the coverage, accuracy, and readability of cancer information from Wikipedia with a peer-reviewed web site (National Cancer Institute's Physician Data Query (PDQ) comprehensive cancer database	Oncology	Coverage, accuracy, and readability (Flesch-Kincaid grade level)
**2**	Tulbert (2011) [199]	Descriptive quality assessment of wiki content paper	Wiki (Wikipedia)	MediaWiki	Online patient education materials were comparatively assessed for readability and length in words	Dermatology	Flesch-Kincaid Grade Level and Flesch Reading Ease Scale
**2**	Wood (2010) [137]	Descriptive quality assessment of wiki content paper	Wiki (Wikipedia)	MediaWiki	Evaluate the medical content of Wikipedia entries about respiratory diseases and assess whether it could contribute to Pathology teaching	Pathology, education	Accuracy, presence of obvious mistakes, and usefulness for pathology teaching
**2**	Wu (2010) (G) [96]	Descriptive quality assessment of wiki content paper	Wiki (Wikipedia) and Google knol	MediaWiki, Google Knol	Comparison of two kinds of online encyclopaedias Wikipedia (consumer-oriented) and	General consumer health	Page views per year, text words, readability, page strength, citation numbers, and citation types

					Google Knol (expert-oriented)		
**3**	Andrus (2010) (A) [151]	Case study	Wiki	wikiacc.org	Nursing education	Nursing, education	Improved quality of work, satisfaction
**3**	Archambault (2011) (G, A) [155]	Survey	Wiki	N/A	Trauma care	Emergency medicine	Barriers and facilitators about the use of wiki-based reminders
**3**	Belt (2011) (G, A) [105]	Case study	Wiki (*IUI-Wiki (www.mijnzorgnet.nl/iui/w/wiki) and IVF-Wiki********)	MijnZorgNet BV (www.mijnzorgnet.nl) [258] (social media platform)	Infertility	Gynaecology	Feasibility of using a wiki as a patient participation tool
**3**	Blakely (2007) (G) [177]	Case study	Wiki (*Wikipedia*)	MediaWiki	Mental health	Consumer health	Report about a pharmaceutical company modifying content in Wikipedia
**3**	Bookstaver (2011) [138]	Quasi-experimental (before and after trial)	Wiki	N/A	Evidence-based medicine teaching	Pharmacy, education	Pre/post-test measurement of the retention of 12 key concepts related to understanding and applying Evidence-based medicine principles; post-test opinion
**3**	Buzzi (2009) [200]	Case study	Wiki (*Wikipedia*)	JAWS screen reader, Accessible Rich Internet Applications	Making Wikipedia usable for the blind	Occupational health	Difficulties interacting with the interface
**3**	Chiarella (2009) [171]	Case study	Wiki (*Dealing with Autism http://4griffin.wetpaint.com*) [257]	Wetpaint	Autism	Mental health	Barriers/facilitators encountered during the creation of a wiki
**3**	Ciesielka (2008) [145]	Case study	Wiki (*614comm: Meadville Collaborative Community Project: http://614comm.pbworks.com*) [259]	PBwiki/PBworks**	Nursing education	Nursing, education	Description of users experience with a wiki
**3**	Cinnamon (2010) (G) [146]	Case study	Google docs	Google Docs	International health	Public health	Utility and feasibility of using free and easy-to-use social media (including Google Docs) tools for injury surveillance in low-resource settings
**3**	Cobus (2009) [139]	Case study	Wiki	Blackboard	Public health teaching	Public health, education	Description of a wiki used by public health students intended for students and professionals as opposed to health consumers

**3**	Collier (2010) [72]	Case study	Wiki	N/A	Nursing education	Nursing education	Comments from students about the use of a wiki
**3**	Cousineau (2009) (G, A) [201]	Case study	Wiki (*Wikipedia-trica*)	N/A	Paediatric department morning report supported by a wiki	Healthcare library science, paediatrics, residency education	Comments about impact of a wiki on attendance at morning report
**3**	Culley (2012) (G) [156]	Case study	Wiki	Blackboard	Graduate nursing education	Nursing Education	Student comments about using a wiki
**3**	Damani (2009) (G, A) [202]	Case study	Wiki	PBworks (premium wiki), Microsoft SharePoint	Healthcare librarians sharing search results for evidence in response to clinical questions	Healthcare library science	Comments about usability of PBworks premium wiki compared to Microsoft SharePoint
**3**	Dhillon (2011) (A) [161]	Case study	Wiki	Google Sites	Musculoskeletal curriculum	Radiology, residency education	Radiology residents' overall impression about a wiki
**3**	Felsen (2010) (A) [175]	Case study	Wiki	PBworks	Disseminating educational materials and coordinating the educational program	Primary care and internal medicine, residency education	Written evaluations by the residents
**3**	Gerber (2010) (A) [176]	Case study	Wiki	MediaWiki	A web-based database for standard operating procedures in cardiac anesthesia	Anesthesia	Wiki usage statistics, evolution of a wiki
**3**	Hamilton (2008) (G, A) [140]	Case study	Wiki	N/A	Evidence-based practice physical therapy course	Physiotherapy education	Comments from a student about the use of a wiki
**3**	Hamm (2009) (A) [147]	Case study	Wiki	N/A	Development of lead poisoning prevention tools	Toxicology	Comments from the participants about the use of a wiki
**3**	Hawkins (2010) [160]	Case study	Wiki	Blackboard	Global health issues	Nursing, education	Comments from students about the use of the course wiki
**3**	Hulbert-	Case study	Wiki	Wolverhampton	Applied	Mental health,	Wiki contributions; post-test

	Williams (2010) [141]			Online Learning Framework (WOLF) platform	psychology class	education	questionnaire
**3**	Ioannis (2011) (A) [107]	Experimental (clinical trial)**	Google Docs	Google Docs	Prevention of cardiovascular disease with a shared online diary	Cardiology	Blood pressure levels, cholesterol levels, smoking status, minutes of physical activity
**3**	Jalali (2009) (A) [178]	Case study	Wiki (Medswiki: www.*medswiki.ca*)	Wikispaces	Medical students using a wiki to share course content	Medical education	Wiki usage statistics and barriers to contributions
**3**	Jones (2010) [154]	Case study	Wiki	LearnJCU (James Cook University virtual learning environment)	Learning environment where on-campus and distance students were able to work together to produce material with a wiki	Social work, education	Student comments regarding the process and outcomes of a wiki assignment (issues and benefits)
**3**	Kardong-Edgren (2009) [148]	Case study	Wiki (*CPR Training Doses Research Site*: http://cprstudy.wetpaint.com)	Wetpaint	Uses of a wiki in nursing research to manage the content of a research team studying the effect of brief teaching on CPR skills	Nursing	Experiences with the use of a wiki
**3**	Kitson-Reynolds (2009) [149]	Case study	Wiki	N/A	Process of enquiry-based learning in midwifery	Midwifery education	Evaluation of a wiki as a course compliment
**3**	Koerner (2011) (G, A) [152]	Quasi-experimental (before and after trial)	Wiki	N/A	Use of wiki to support a paediatric elective	Paediatrics, medical education	Pre and post satisfaction survey was administered to assess perceptions of a wiki and its ability to enhance learning
**3**	Kraft (2009) (G, A) [203]	Case study	Wiki	N/A	Providing employees of a	Healthcare library science	Comments from clinicians involved in a pilot test and wiki usage

					large regional health system access to library resources on and off campus		statistics from Google Analytics
**3**	Krebs (2009) (A) [181]	Survey	Wiki (Neuroanatomy at UBC: www.neuroanatomy.ca) [204]	WebCT learning management system at University of British-Columbia	Wiki created as a reference database for students	Medical education	Students evaluations of the use and usefulness of a wiki
**3**	Lanning (2010) [158]	Case study	Wiki (*Palliative Care Resources for Physician Assistants*: http://palliativecareforpas.wetpaint.com) [205]	Wetpaint (www.wetpaint.com)	End-of-life/palliative education in physician assistant programs	Physician assistant, education	Student experiences with a wiki
**3**	Lauber (2009) [180]	Case study	Wiki (*ATEP Wiki*)*****	PBworks	Athletic training education programs are exploring wikis to train Approved Clinical Instructors	Sports medicine and therapy, education	Instructor feedback after using a wiki
**3**	Llambí (2011) [142]	Quasi-experimental (before-and after trial)	Wiki	N/A	Continuing medical education of physicians to help patients stop smoking	Primary care and internal medicine, CME	Pre/post-test measurements of the skills of teaching tobacco cessation; percentage of physicians passing the tobacco cessation skills test
**3**	Ma (2008) [102]	Case study	Hybrid wiki (*OrthoChina*: www.orthochina.com) [206]	Custom-built application******	Continuing medical education about musculoskeletal disorders	Orthopaedics, CME	Wiki usage statistics and qualitative description of content
**3**	Matlin (2009) (G, A) [207]	Case study	Wiki	Wetpaint	Sharing of ressources to improve student researching skills	Pharmacy, education	Authors experience with wiki
**3**	Meenan (2009) [166]	Case study	Wiki (RadIT)*******	MediaWiki	Internal knowledge	Radiology	Wiki usage statistics

					management system sharing various informatics support tools within a radiology department		
**3**	Miller (2009) [159]	Case study	Wiki	Google Sites	During an advanced pharmacy practice rotation in acute care setting, wikis were used to store questions and answers that arise during rounds	Pharmacy, education	Survey of student opinions about wiki use
**3**	Mirk (2010) [162]	Quasi-experimental (before and after trial)	Wiki	Wetpaint	Pharmacy students enrolled in a course called "Landmark trials in primary care" to learn how to apply evidence in practice	Pharmacy, education	Pre/post evaluation of students' attitudes, level of perceived usefulness, degree of involvement with wiki collaboration, and level of satisfaction with wiki collaboration (5-point Likert scale)
**3**	Moeller (2010) [108]	Experimental (RCT)	Wiki	N/A	Comparison of three interactive components (wiki, chat and interactive diagnostic context) on learning, aspects in the context of a problem-based learning	Medical Education	Self-administered questionnaires: learning effect, communication; collaboration; student satisfaction; diagnostic approach; realism;
**3**	Moen (2009) [106]	Case study	Wiki (RareICT: http://goo.gl/07MHm) [208]	MinJournal.no is a social media platform	Create a collaborative environment for	General health information	Experiences and feedback from participants

				developed by the University of Oslo and several major hospitals in Norway [208]	peer support and knowledge construction related to a rare anorectal anomaly		
**3**	Montano (2010) [170]	Case study	Wiki (WIKINVESTIGACION: www.wikinvestigacion.org) [209]	Wikispaces	A community of researchers developed a wiki for the sharing and development learning and investigation resources in a hospital setting	Healthcare library science	Needs of the research community; opportunities provided by Web 2.0 tools; definition of the spaces that would be developed: elements, members and different access levels
**3**	Morley (2011) [153]	Case study	Wiki	University of Bournemouth's virtual learning environment (myBU); Blackboard	Wikis were introduced in a virtual learning environment to support on ongoing sociology of health course	Nursing, Education	Students' online contributions; students' and teachers' comments
**3**	Morose (2007)(D) [167]	Descriptive quality assessment of wiki content paper	Wiki (Participatory ergonomics: 2 www.cre-msd.uwaterloo.ca/participatoryergonomics)********	Wiki was developed on Centre of Research Expertise for the Prevention of Musculoskeletal Disorders website: www.cre-msd.uwaterloo.ca [210]	Developing a participatory ergonomics website to summarize and share important information	Ergonomics/workers safety	Wiki usage statistics; exit questionnaire to explore: the dissemination of information using a wiki; the decision-making process based on the quality of information in a wiki; and reasons for contributing to a wiki
**3**	Moser (2011) (G, A) [165]	Case study	Wiki	N/A	Wiki used to get feedback from stakeholders about measures to assess determinants of health	Primary care	Lessons learned from a natural experiment
**3**	Mosquera	Case study	Google docs	Google docs	Veterans' health	General	Report about a HIPAA security

	(2010) (G) [179]					healthcare	breach because physicians and medical students used Google Docs to enter notes about patient care
**3**	Muir (2010) (A) [168]	Case study	Wiki (*UCLA Radiology Residents*: http://pediatricimaging.wikispaces.com) [211]	Wikispaces	Wiki used by residents to share interesting cases	Radiology, residency education	Comments about experience using a wiki
**3**	Musil (2011) (G, A) [150]	Case study	Wiki	N/A	A wiki was used as a virtual learning environment	Pharmacy and social sciences, education	Students' comments about the use of a wiki
**3**	Phadtare (2009) [27]	Experimental (RCT)	Google Docs	Google Docs	Google docs was used to teach scientific writing skills	Healthcare Education	Manuscript quality (Six-Subgroup Quality Scale); satisfaction; post hoc number of communication events (emails or phone calls) between participants and mentors
**3**	Philip (2008) [143]	Observational analytic design	Wiki	MediaWiki	Reverse teaching methodology in which students are given disease diagnosis and then asked to construct a patient case on a wiki to learn about clinical anatomy	Medical Education	Student confidence (7-point Likert scale) about different clinical skills; absolute increase in percentage of students feeling confident (more than 4 on Likert scale)
**3**	Powers (2009) (G, A) [212]	Case study	Wiki	PBwiki	Wiki used to enable students to identify and gain access to relevant educational resources	Osteopathy, Healthcare library science, education	Comments about using a wiki
**3**	Seebregts (2009) [76]	Case study	Wiki (OpenMRS Wiki: https://wiki.openmrs.org) [213]	MediaWIki; since 2010, OpenMRS Wiki is powered by Open Source Atlassian Confluence team	OpenMRS Implementers have created a wiki to share resources about implementing	Public health	Discussion about the benefits of using a wiki

				collaboration software (www.atlassian.com)	OpenMRS (an open source electronic medical record mainly used for HIV patients in Africa)		
**3**	Shaw (2010) [144]	Case study	Wiki (Marshall-Smith Wiki: www.marshallsmith.org) [214] *******	N/A	Description of a wiki to facilitate data collection and sharing about the phenotype and natural history of the rare Marshall-Smith Syndrome	Medical genetics	Discussion about the benefits of using wiki
**3**	Steininger (2010) [174]	Survey	Wiki	N/A	Web 2.0 portal supervised by medical experts to disseminate credible information to consumers	Primary care and internal medicine	Assessment of patients' and physicians' needs; willingness to participate in development
**3**	Streeter (2007) [169]	Case study	Wiki (RadiologyWiki: www.RadiologyWiki.org) [262]	MediaWiki	Creation of a dynamic online radiology educational resource	Radiology, education	Discussion about challenges and benefits of creating RadiologyWiki
**3**	Stutsky (2009) (D) [109]	Experimental (RCT)	Wiki	PBwiki (http://pbwiki.com) [215] *********	N/A	Nursing	Empowerment: CWEQ-II (Conditions of Work Effectiveness Questionnaire-II); Psychological Empowerment Instrument; Measuring Leadership Practices (The Leadership Practices Inventory (LPI))
**3**	Umland (2011) (G) [157]	Case study	Wiki	N/A	Women's health course in pharmacy curriculum	Pharmacy, education	Student evaluations about the usefulness of a wiki
**3**	Van Der Schoor-Knijnenburg	Case study	Wiki (FreyaWIKI: www.freyawiki.nl) [216]	Custom-built application	Production of a multidisciplinary guideline for	Gynecology	Perceived benefits expressed by author

	(2009) [103]				subfertility care involving patients		
**3**	Varga-Atkins (2010) [32]	Case study	Wiki (PPD wiki)*****	University of Liverpool virtual learning environment with Teams LX wiki (Blackboard)	Problem-based learning course added to a wiki to teach professionalism	Medical Education	Student and facilitator views about wiki use
**3**	Wan (2009) (D) [101]	Case study	Hybrid wiki (Wikibreathe tool/OCTAPUS: http://knowledgetranslation.ca/octapus/login.php)	Custom-built application***	Reverse engineering of content as a task for finding usability problems using the Wikibreathe tool to develop an asthma action plan	Respirology	Systems usability scale (ten-item scale each using a 5-point Likert scale giving a global view of usability); user (patients, clinicians) attitudes towards the tool
**3**	Welsh (2007) [163]	Case study	Wiki (DrugScope Procedure Wiki)*****	N/A	Internal wiki used as content management system to share procedures, self-guided training and tacit knowledge	Pharmacy	Author's experience with using a wiki
**3**	Wright (2009) [172]	Case study	Wiki (Clinfowiki: www.clinfowiki.org) [217]	MediaWiki	To provide clinical informaticians a place to share experiences and clinical decision support content	Medical informatics	Wiki usage statistics
**3**	Wu (2009) (G) [74]	Quasi-experimental (before and after trial)	Wiki	N/A	Wiki-supported course teaching technology and information skills for nursing students	Healthcare library and nursing, education	Pre/post information literacy levels and students' opinions
**3**	Yates (2011) [164]	Case study	Wiki (Intelink: www.intelink.gov [31]; and	MediaWiki (Intelink) and	US agencies employed wikis	Disaster management	Perceived benefits and challenges related to using wiki

			U.S. Air Force Chief of Staff's Crisis Action Team (AFCAT) SharePoint site*******)	Microsoft SharePoint (AFCAT site)	as the main knowledge management system to organise disaster response in Haiti Earthquake		
**3**	Yu (2011) (A) [173]	Case study	Wiki (Beth Israel Deaconess Medical Center hospitalist wiki site*******)	Microsoft SharePoint	A closed and secure wiki was used as a content management system to share information in a hospitalist group	Primary care and internal medicine	Wiki usage statistics and key elements for success

Notes:

*** G=Grey literature; A=Abstract; D= Dissertation**

** PBworks was formally known as PBwiki.

** Methods for this abstract are very poorly described. It is impossible to determine if the paper was randomized or not. All that is mentioned is that it was a clinical trial.

*** This application was developed with the following software: jQuery, version 1.3.1 (a JavaScript library with built-in AJAX functions was used for the client-side interaction); wkpdf, version 0.2 (used for PDF generation); PHP, version 5.2 (including PEAR and MDB2) (used for server-side functionality); and MySQL, version 5.1 (used for databases).

**** Both Wikipedia and WikiHealth are powered by MediaWiki.

***** URL was not found.

****** OrthoChina was built to guard against copyright infringement and maintain safety of the operating system, the author selected free open-source applications for the operation of OrthoChina: Redhat Linux AS2 (operating system; Redhat Inc., Beijing, China, www.redhat.com.cn) and MySQL (database; of MySQL AB, Cupertino, CA, http://www.mysql.com) in conjunction with Tomcat-connector, Apache and Tomcat (Apache Software Foundation, Forest Hill,MD, http://www.apache.org). The Web site is designed and structured using a modified wiki concept. 1) Web content is created collaboratively by users through an Internet browser. 2) Information may be posted, edited, deleted, and updated by anyone with permission to do so, at any time. 1) The moderator can post content in any page, but can only edit, delete, and update the content in the specific pages he moderates. 2) The other users can only post content in forums (pages open for discussion), and can only edit and update the content posted personally, but they cannot delete the content once posted. 3) All content is open to viewing, and is monitored by the general orthopaedic users. 4) All editing, deleting, updating, credit score increasing and decreasing steps are tracked in detail, such as time, IP address, user, etc.

******* No URL could be found. This wiki is closed and password protected.

******** URL is no longer active.

********** Platinum level PBwiki (now PBworks): allows for enterprise-grade encryption on all Web pages)

********** **List of 52 wiki assessed in Dobrogowska-Schlebusch paper, URL and software used**

Ask Dr Wiki [218]	http://askdrwiki.com/	MediaWiki
Clinfowiki [217]	http://www.clinfowiki.org/wiki/index.php/Main_Page	MediaWiki
Billingwiki	n/a	n/a
Consumer Health Information Service [219]	http://chis.wikidot.com/about	Wikidot (www.wikidot.com)
Demystifying Depression [220]	http://en.wikibooks.org/wiki/Demystifying_Depression	MediaWiki
Diabetes Wiki [221]	http://diabetes.wikia.com/wiki/Diabetes_Wiki	Wikia (MediaWiki)
Diagnostic Radiology [222]	http://en.wikibooks.org/wiki/Diagnostic_Radiology	MediaWiki
DocCheck Flexicon [223]	http://flexikon.doccheck.com/en/Special:Mainpage	n/a
EBM Librarian [224]	https://sites.google.com/site/ebmlibrarian/home	Google Sites (previously on Wetpaint)
Emergency Medicine [225]	http://en.wikibooks.org/wiki/Emergency_Medicine	MediaWiki
Flu Wiki [226]	http://www.fluwikie.com/&refdoi=10.1186/1472-6920-6-41 (http://www.newfluwiki2.com)	SoapBlox
Ganfyd [30]	http://www.ganfyd.org	MediaWiki
Handbook of genetic counselling [227]	http://en.wikibooks.org/wiki/Handbook_of_Genetic_Counseling	MediaWiki
HealthGrid Wiki [228]	http://wiki.healthgrid.org/Main_Page	MediaWiki
Immunology [229]	http://en.wikibooks.org/wiki/Immunology	MediaWiki
Wikikidney [230]	http://www.wikikidney.org/index.php?title=Main_Page	MediaWiki
Medical Imaging [231]	http://medicalimaging.wikia.com/wiki/Main_Page	Wikia (MediaWiki)
Mla-hls [232]	http://mla-hls.wikispaces.com	Wikispaces
Mighealth (Migrant health) [233]	http://www.mighealth.net/uk/index.php/Main_Page	MediaWiki
ECGpedia [234]	http://en.ecgpedia.org/wiki/Main_Page	MediaWiki
The McGill Global Health Resource Guide [235]	http://wikisites.mcgill.ca/GlobalHealthGuide/index.php/Main_Page	MediaWiki
EBHC [236]	http://ebhcstrategies.wetpaint.com	Wetpaint
CHI TPL	n/a	n/a
Neurodegeneration Research Wiki [237]	http://wiki.iop.kcl.ac.uk/default.aspx/Neurodegeneration/Neurodegeneration%20Research%20Wiki.html	n/a
Pharm Lib [238]	http://pharmlib.pbworks.com/w/page/16284404/FrontPage	PBworks
PubDrug	n/a	
Quality of Medical Data [239]	http://medicaldata.wikia.com/wiki/Main_Page	MediaWiki (wikia)
Radiation Oncology [240]	http://en.wikibooks.org/wiki/Radiation_Oncology	MediaWiki
RadiologyWiki [262]	http://www.radiologywiki.org/wiki	MediaWiki
Radiopaedia [241]	http://radiopaedia.org	TrikeApps (http://trikeapps.com/projects/powerful/radiopaedia) (Radiopaedia was previously on MediaWiki)
RadsWiki	n/a	n/a
Rosacea Wikibook [242]	http://en.wikibooks.org/wiki/Rosacea	MediaWiki

Wikiversity School:Medicine [243]	http://en.wikiversity.org/wiki/School:Medicine	MediaWiki
Street Medic Wikia [244]	http://medic.wikia.com/wiki/Main_Page	MediaWiki (wikia)
Surgery [245]	http://en.wikibooks.org/wiki/Surgery	MediaWiki
UBC HealthLib Wiki (HLWIKI) [246]	http://hlwiki.slais.ubc.ca/index.php/UBC_HealthLib-Wiki_-_A_Knowledge-Base_for_Health_Librarians	MediaWiki
WebHealth [247]	http://webhealth.com/about/	WordPress (http://wordpress.com/)
Wellness Wiki [248]	http://wellness.wikispaces.com	Wikispaces
WikiCancer [249]	http://www.wikicancer.org	Wetpaint
WikiEcho [97]	http://www.wikiecho.org/wiki/Main_Page	MediaWiki and Semantic MediaWiki (http://www.semantic-mediawiki.org/wiki/Semantic_MediaWiki)
WikiHealth [260]	http://www.wikihealth.com/Main_Page	MediaWiki
WikiHealthCare [250]	http://wikihealthcare.jointcommission.org/bin/view/Home/WebHome	TWiki
WikiMD	http://www.wikimd.org/index.php/Main_Page (broken link)	MediaWiki
Wikisurgery	http://www.wikisurgery.com (broken link)	MediaWiki
WiserWiki [251]	http://www.elsevier.com/about/press-releases/health-sciences/elseviers-wiserwiki-allows-physicians-to-update-evidence-based-medical-information-with-experience-based-practice-insights	n/a
Nursing Wiki (Pflegewiki) [252]	http://www.pflegewiki.de/wiki/Hauptseite	MediaWiki
OpenWetWare [38]	http://openwetware.org/wiki/Main_Page	MediaWiki
OncoWiki [256]	http://oncowiki.info/index.php?title=Main_Page	MediaWiki
Orthopaedic Surgery [253]	http://en.wikibooks.org/wiki/Orthopaedic_Surgery	MediaWiki
Human Physiology [254]	http://en.wikibooks.org/wiki/Human_Physiology	MediaWiki
MUSC	n/a	
Pharmacology [255]	http://en.wikibooks.org/wiki/Pharmacology	MediaWiki
Wiki Doc [98]	http://www.wikidoc.org/index.php/Main_Page	MediaWiki and Semantic MediaWiki (http://www.semantic-mediawiki.org/wiki/Semantic_MediaWiki)
