# Supplementary material for: Wikis and Collaborative Writing Applications in Health Care: A Scoping Review
Source: J Med Internet Res. 2013 Oct 8;15(10):e210. doi: 10.2196/jmir.2787 (PMC3929050; doi:10.2196/jmir.2787)
Supplement: Supplementary file 2 [file jmir_v15i10e210_app2.pdf]

Author (Year of publication) Country	Characteristics of population studied	Response rate (%) (Respondents (N)/size (N) of total population)	Results	Time of assessment of use	Contribution rate	Prevalence of use	Reason for CWA use
**CWA use in specific projects**							
Gupta (2010) [263] Canada	18 Healthcare professionals (Respirologists, Primary care physicians and Asthma Educators) and 21 patients	90% (35/39)	The proportion of use is not presented; Participants logged in 4.4, 5.8, 6.4, and 7.1 mean times/week; for 14, 16, 37, and 25 mean minutes/day; making 6.3, 6.0, 7.2, and 9.3 mean changes/day; 77% (27/35) found the tool effective	1 week period (year not specified)	N/A	N/A	A wiki; like tool was used to seek multiple; stakeholder input and agreement about the visual aspect of an Asthma Action Plan
Kohli (2011) [53] USA	Radiology residents in a single radiology residency program	85% (51/60)	Wiki visits (mean): 5.6 times a week; Know how to edit wiki: 78% (n=40); Know how to add new page: 37% (n=19); Plan to add content to a wiki in the future: 71% (n=36)	3 year period (year not specified)	69% (n=35)	100% (n=51)	A wiki was used as a knowledge management system to support residents' work on a daily basis (schedules, phone numbers, dictation templates, rotation/call information, educational content)
Williams (2011) [110] Australia	Undergraduate paramedic students (2nd year); Male/Female: 9/20; Age: 21 respondents were 25 years old or less	49% (29/59)	Recommend using wiki in future courses: 41% (n=12); 41% (n=12) were neutral for this aspect; 14% (n=4) did not recommend its future use	N/A	N/A	N/A	A wiki was used to support case; based learning in a course given to paramedic undergraduate students. The wiki supported the blended approach to case; based learning using a mix of face; to; face and e; learning asynchronous

							communication between the students. The course wiki seemed to be an ideal way to promote cooperative learning, the sharing of ideas and the joint of development of common artefacts.
**CWA use in general (including Wikipedia)**							
Alkhateeb (2011) [119] USA	Pharmacists in West Virginia attending a conference	100% (50/50) (note: all pharmacists attending a conference)	The vast majority of respondents reported using at least one type of social media tool, with the most frequently used applications including: YouTube (74%), Wikipedia (72%) and Facebook (50%).	2009 (October)	N/A	72%	This study describes social media use among pharmacists. Use was mainly for personal reasons.
Archambault (2010) [29] (G) Canada	Fifth; year residents enrolled in a Royal College of Physicians and Surgeons of Canada training program in Emergency Medicine in Canada	71 residents had access to a Google Docs slideshow	102 landmark articles had been summarized and critically reviewed	2009	22% (16/71)	N/A	Google docs was used to update a summary of landmark articles in Emergency Medicine
Bender (2011) [130] (G) Canada	Scholars and the editors of the Open Medicine Wiki Asynchronous Telehealth Scoping Review: 12 editors registered with the wiki (5 team members and 7 other non; team members). There is only information on 4/7 of the non; team members (male: 3; Canadians: 3; age: 30 to 60 y; previous use of a wiki: 4; University; affiliated: 3; health researchers: 2; health administrator: 1;	4/7 non; team members completed the registration questionnaire	1222 visits to the wiki, 3996 page views, 875 unique visitors, 5 visitors submitted a total of 6 contributions: 3 contributions were made to the article itself, and 3 to the discussion pages. None of the contributions enhanced the evidence base of the scoping review. User accounts: 61; “Genuine” user accounts: 13 “Fake” accounts: 21 created by either automated scripts or individuals entering malicious information that was either false or nonsensical.	2009 (June 9) to 2010 (April 10)	0.57% (n=5)	N/A	A scoping review was published on the Open Medicine Wiki to facilitate its updating by all readers

	health practitioner: 1; previous contribution to a wiki: 1; conflict of interest: 1). Visitors came from 66 different countries, with 72.2% of visits originating from Canada or the United States						
Brokowski (2009) [116] USA	Pharmacists from 78 different U.S. pharmacy associations contacted using a mailing list; mean age: 48 years (range 23–86 y); Male/Female ratio: 44%/66%; mean experience 23 years (range 0–65 y); Degree: B.Sc. (52%, n=549), PharmD (40%, n=422), Other (e.g., PhD, MSc.) (9%, n=95); Residency training (22%)	3% (1056/38110)	19% (69/369) respond that they trusted Wikipedia; 12% (43/369) would recommend Wikipedia to other pharmacists; 7% (25/369) would recommend Wikipedia to consumers/patients; 28% reported using it to obtain drug information; 28% of the respondents who reported using Wikipedia to obtain drug information were familiar with who edits and manages the Web site.	2009 (February 2; March 14)	N/A	35% (369/1056)	Wikipedia was used to obtain medication indications and drug information
Dodson (2011) [111] USA	Librarians in health science or/and hospital	N/A (10 respondents)	The most common department that librarians reported supporting with blogs or wikis was the medicine department, followed by pediatrics and family medicine.	2009	N/A	N/A	Wikis were used to support patient care and educational activities of medical departments (medicine, pediatrics and family medicine).
Gonzalez de Dios (2011) [120] Spain	Pediatric neurologists and residents attending a conference	100% (44/44) (36 pediatric neurologists, 8 residents)	91% of participants did not use wikis, 7% used them seldomly, and 2% used them often. 84% did not use Google Docs, 16% used it seldomly, 0% used it often.	2010 (October)	N/A	9% (wikis); 16% (Google Docs)	This paper surveyed the types of web and web2.0 resources used by clinicians
Harris (2010) [112] USA	1st year psychology students	N/A (Note: no information on the general population.) (271 respondents)	First year students used information obtained from Wikipedia; 36% (n=97) used Wikipedia information for research papers, presentations, and other course requirements; 63%	N/A	N/A	64% (n=173)	This paper explores how Wikipedia is used by 1st year psychology students. Students use it as

			(n=171) never, rarely, or occasionally attempted to verify information obtained from Wikipedia by checking other sources.				a source of general information and for academic purposes.
Hickerson (2009) [122] (G) USA	Users of Wikipedia (n=45) and WikiHealth (n=16) were surveyed online about their use of these wikis compared to none; wiki sites. College training (>4y): 38% (n=25) Postgraduate degree: 41% (n=27). Income more than $75,000 (US)/year: 43% (n=28). Caucasian: 80% (n=52). Male: 68% (n=44). Hours spent on the Internet (>21h/week): 47% (n=31). Hours spent on the wiki (>5h/week): 49% (n=32)	Response rate unknown; 65 respondents	More participants were committed to using a wiki in the future compared to none; wiki sites. The overall dialogic scores for the two wikis were also found to have positive and significant correlations to finding the website valuable and users' commitment to future use. [a]	2 month period (year unknown)	N/A	N/A	Both wikis (Wikipedia and Wikihealth) were used for general health information.
Hughes (2009) [42] UK	35 junior physicians were selected via stratified sampling of 300 graduates from a London medical school (to ensure adequate representation of top 10 specialties); mean age: 27 years; Male/Female ratio: 0.75:1; physicians were post; graduate year 2 or 3 (Foundation year 2 or Specialist training year 1)	63% (35/55)	Junior physicians used wikis in their medical practice; Junior physicians used Wikipedia to find medical information; Few junior physicians made regular contributions to a medical wiki site; main reason for using Web2.0 sites: ease of use: 93% (33/35); main barrier against using Web2.0: limits in quality of information found (trustworthiness): 72% (27/35)	2008 (July)	3% (1/35)	80% (wikis) (28/35); 70% (Wikipedia) (25/35)	This paper identified the junior physician's reasons to use Web2.0 tools (including wikis): ease of use, quality of information (up; to; date compared to textbooks, broader scope, contained interactive images), to solve an immediate defined clinical problem and for background reading on a subject.
Iyer (2011) [117] USA	43 healthcare professionals (35 physicians, 7 physician	51% (79/154)	Healthcare professionals and pharmacists reported the use of Google or Wikipedia at least	2009 (March 6th ; April	N/A	18% (n=8 healthcare professional	This paper attempts to determine where

	assistants, 6 residents, 1 nurse practitioner) and 36 pharmacists working in the community clinics of one university network; mean age (SD) for clinicians was 43.23 (9.52) years and for pharmacists was 40.19 (10.60) years; Female/Male ratio: clinicians: 60%/40%; pharmacists: 61%/39%; years of experience (SD): clinicians 11.76 (9.48) and pharmacists: 13.02 (10.09)		daily (note: impossible to differentiate). The top sources used at least daily for seeking drug information among: A) healthcare professionals: (1) drug information databases (46%; n=20) (e.g. Micromedex and ePocrates); (2) hand; held devices (23%; n=10); (3) other online sources [Google or Wikipedia] (19%; n=8); (4) medical literature indices [PubMed, Medline, and CINAHL] (14%, n=6). B) pharmacists: (1) drug information databases (78%; n=28) (e.g. Micromedex and ePocrates); (2) other online sources [Google or Wikipedia] (28%; n=10); (3) medical literature indices [PubMed, Medline, and CINAHL] (19%, n=7); (4) hand; held devices (11%; n=4) .	29th)		s); 28% (n=10 pharmacists)	healthcare professionals obtain drug information for clinical care and to stay updated on the latest drugs
Judd (2010) [123] Australia	Undergraduate medical and biomedical students	No response rate[b]	Google was the most popular information seeking site: students' usage increased from 24% (n=1200) in 2005 to 31% (n=1550) in 2009; Wikipedia use increased between 2005 and 2009; Use of NIH sites (PubMed, MedlinePlus and the National Library of Medicine portals) declined from 8% (n=400) in 2005 to 4% (n=200) in 2009	2005 ; 2009	N/A	2005: 2% (n=100); 2009: 16% (n=800)	Wikipedia was used to find biomedical information
Judd (2011) [124] Australia	Undergraduate medical and biomedical students; Computer session logs (n=620) of 1st; , 2nd; and 3rd; year medical students' biomedical searches in an open; access computer laboratory	No response rate[c]	Website use for biomedical searches: 1 Google (69.8%, n=433); 2 Wikipedia (51.0%, n=316); 3 eMedicine (21.5%, n=133); 4 NIH (16.5%, n=102); 5; University's Library (13.4%, n=83). Students' Wikipedia use decreased depending on their training level (p<0.001)	2007 (April/May)	N/A	Overall: 51%; 1st year students: 70.2% (132/188); 2nd year students: 49.5% (98/198); 3rd year	Wikipedia was used to find biomedical information

						students: 36.8% (86/234)	
Laurent (2009) [125] UK	N/A[d]	N/A	Wikipedia ranked among the first ten results in 71; 85% of search engines and keywords tested. Wikipedia surpassed MedlinePlus and NHS Direct Online (except for queries from the latter on Google UK). Wikipedia ranked highest for rare diseases. Wikipedia articles were viewed more often than MedlinePlus Topic pages (p=0.001) but for MedlinePlus Encyclopedia pages, the trend was not significant (p=0.07, Jan 2008) and (p=0.10, June 2008).	2008 (Aug 19; 23 and Sept 12; 13)	N/A	N/A	Wikipedia was used to find general health information
Law (2011) [126] USA	Consumers	No response rate.[e]	For generic drugs, Wikipedia is the first result for: 84.9% (236/278) Google.ca searches; 84.2% (234/278) Bing searches; 86.3% (240/278) Yahoo searches; 21.6% (60/278) Google.com searches. The National Library of Medicine is the first result for 74.8% (208/278) Google.com searches. For brand name drugs, Wikipedia is the first result for: 1% (2/198) Bing searches; 1% (2/198) Google.ca searches; 1% (2/198) Yahoo searches; 0.5% (1/198) Google.com searches. The National Library of Medicine is the first result for 71.7% (142/198) Google.com searches. Drug.com is the first result for 54.5% (108/198) Bing/Google.ca/Yahoo/Google .com searches.	2010 (June)	N/A	N/A	Wikipedia used as a source of drug information.
Lemley (2009) [113]	Medical school educators and Nursing school	3% (55/1679) (36 Medical school	The most common Web 2.0 tools used in the curricula of	N/A	N/A	N/A	Using Web2.0

USA	educators (using mailing lists from different organizations: DR; ED, American Association of Colleges of Nursing Instructional Leadership Network, Association of Academic Health Sciences Libraries)	educators and 19 nursing school educators)	medical schools (in order from most frequent to least frequent): 1 none, 2 podcasting, 3 videocasting, 4 wikis, 5 blogs, 6 Flickr, 7 YouTube, 8 MySpace/Facebook, 9 Moodle. In nursing schools the most frequent Web2.0 tools are (in order from most frequent to least frequent): 1 none, 2 podcasts, 3 videocasts, 4 wikis, 5 blog, 6 Moodle, 7 Flick.				tools for teaching
Limdi (2011) [128] UK	104 consecutive patients consulting an Inflammatory bowel disease clinic; age range: 45 to 64; Male/Female ratio: 46%/54%; highest educational level: high school/comprehensive (46.2%, (n=48)), sixth form/technical college: (14.4%, n=15), university graduate (32.7%, n=34), postgraduate (4.8%, n=5)	100% (104/104)	The most popular site was Crohn's and Colitis UK (n=24) with 22 "useful" and 0 "poor quality" ratings. Wikipedia was second (n=21) with 13 "useful" and 5 "poor quality" ratings.	N/A	N/A	N/A	Wikipedia was used to find medical information about Inflammatory Bowel Disease
Martin (2011) [127] (G) USA	14 first; year pharmaceutical students	100% (14/14)	Typical search strategy was first using Wikipedia, then PubMed and then MD Consult	N/A	N/A	N/A	Wikipedia was used to find relevant biomedical and pharmaceutical information.
Sandars (2007; a) [114] UK	Medical students and psychology students	Response rate unknown; first year medical students (n=197) and psychology students (n=80)	Use of a blog or a wiki was not differentiated. Intention to use blogs/wikis in the future: yes: 5% (n=19); maybe: 42% (n=116) ; no: 26% (n=72). Rate of authoring a blog/wiki was not differentiated; Intention to author a blog/wiki in the future: yes: 5% (n=19); no: 37% (n=102); maybe: 35% (n=97)	N/A	14% (n=38)	29% (n=80)	Using blogs or wikis for academic learning

Sandars (2007; b) [189] UK	Medical students and qualified medical practitioners members of British Medical Association Consultants (n=389), GP (n=96), Doctors in training (n=64), Medical students (n=593); Mean age of: (1) Consultant: 48.3, (2) GP: 42.3, (3) Doctors in training: 37.8, (4) Medical students: 24.4; Gender: Male: 49.6% (n=567), Female: 50.4% (n=575)	21% (1239/5889) (note: 6000 emails were sent but 111 did not work)	Familiarity with wikis: Consultant: 68.9% (n=268); GP: 59.4% (n=57); Doctors in training: 79.7% (n=51); Medical students: 72% (n=427)	2007 (July)	N/A	Consultants: 55.3% (n=215);<br><br>GPs: 50.4% (n=58); Doctors in training: 57.8% (n=37); Students: 80.9% (n=480)	Using wikis for personal or educational use
Sandars (2008) [115] UK	All first; year medical students at Leeds University; Gender: Female: 67%, (n=142)/Male: 33% (n=70); age (mean): 19 y (range 17; 32, 90% < 21)	92% (195/212)	Previous contributions to a wiki: Male: 18% (11/65); Female: 2% (3/129); p < 0.001 (gender difference)	2006 (October)	7.2%	N/A	Wikis used for e; learning
Santos (2007) [129] Spain	Urology patients. Age: 60.98 (SE 15.08) Gender: Male: 81.6% / Female: 18.4%	1062 respondents (note: 1111 questionnaires were received; 49 questionnaires were not usable)	Wikipedia was the fourth most visited website (among other Spanish; language sites).	2006 (September to December)	N/A	N/A	Wikipedia used for health information about urology problems
Schweitzer (2008) [121] USA	This was a two part study among university psychology students: Part 1: First year psychology students; age (mean): 19.1 years; Female 54.7%/ Male 45.3%; Part 2: Senior psychology students (majors and 4th year students); age (mean): 23.5 years; Female 58.9%/ Male 41.1%;	Part 1: 38% (918/2400) Part 2: N/A (76 respondents; no information on the overall population)	Part 1: Familiarity with Wikipedia: 18.8% (n=173) had never heard of it; 17.6% (n=162) had heard of it 63,6% (n=584) had used it in the past 18.5% (n=170) reported using it on a regular basis. Frequency of: 1) personal use: regularly: 14.9% (n=137); once or twice: 41.7% (n=383); never: 43.4% (n=398); 2) use for high school paper: regularly: 16.9% (n=88); once or twice: 39.8% (n=365); never: 43.3% (n=397); 3) use for college paper: regularly: 4.1% (n=38); once or twice: 14% (n=129); never: 82% (n=753); 4) Wikipedia use as a	N/A	6.1%	1st year students' personal regular use: 14.9% ; 18.5%;<br><br>1st year students' regular use for college paper: 4.1%; 1st year students' regular use of Wikipedia as a citation: 2.4%; 1st year	This paper assesses the prior use of Wikipedia by psychology students. Among stated uses were for personal use, school related writing projects and as formal reference in academic work.

			reference in college paper: regularly: 2.4% (n=22); once or twice: 6.3% (n=58); never: 91.3% (n=838);5) reference to Wikipedia in psychology project: regularly: 0.6% (n=6); once or twice: 2.4% (n=22); never 97% (n=890). Part 2: Familiarity with Wikipedia: 14.5% (n=133) had never heard of it; 21.1% (n=194) had seen it but used rarely; 64.5% (n=592) were regular users; 28.9% (n=265) used it on a frequent basis. Reasons for Wikipedia use: 80.3% (n=737) out of curiosity; school related paper 77.3% (n=710); psychology specific paper: 43.1% (n=396); never cited Wikipedia: 39.4% (n=362); had edited Wikipedia: 6.1% (n=56)			students' regular use of Wikipedia as a citation for a psychology paper: 0.6%; Senior students regular use: 64.5%; Senior students' use of Wikipedia for a school related paper: 77.3%; Senior student use for a psychology paper: 43.1%	
Usher (2011) [118] Australia	935 Healthcare professionals; Psychiatrists: 1% (n=11), GPs: 11% (n=104), Social Workers: 12% (n=109), Dieticians: 14% (n=134),Chiropractors: 2% (n=15), Physiotherapists: 29% (n=271), Optometrists: 14% (n=128), Pharmacists: 17% (n=163); age: <30: 22.7% (n=212), 30; 50: 49.1% (n=459), >50: 28.2% (n=264); Clinical experience: <10 y: 33% (n=309); >10 y: 67% (n=626). Gender: Male: 38.5% (n=360)/Female: 61.5% (n=575). Practice type: Private practice: 64.7% (n=605);	Response rate unknown; 935 respondents answered online survey (note: 1,085 responses were collected; 150 responses excluded because of missing data)	Overall, 9.5% (n=89) of healthcare professionals stated that they used social media to deliver care to patients; 19.1% (n=179) stated that they would use social media for personal purposes only, and 71.3% (n=667) stated that they would not use social media at all. Ranking of different social media use for healthcare delivery based on the frequency of use in the last 12 months: 1 email; 2 Skype; 3 iPhone; 4 Facebook; 5 Twitter; 6 instant messaging; 7 message boards; 8 chat rooms; 9 blogs; 10 wikis 11 MySpace; 12 YouTube. Ranking of social media use for personal reasons based on the frequency of use in the last 12 months: 1 email; 2	2009	N/A	N/A	Use of Web2.0 tools for Healthcare delivery and personal needs

	Government: 26% (n=244); Location: Major city: 57.4% (n=537); Inner regional 18.2% (n=170); Outer regional 18.8% (n=176); remote 5.6% (n=52)		Facebook; 3 iPhone; 4 Skype; 5 Twitter; 6 wikis; 7 instant messaging; 8 blogs; 9 YouTube; 10 message boards; 11 chat rooms; 12 MySpace; 13 Medworm; 14 Flickr				

G = Grey literature

a. The dialogical score is a composite score based on a summary of the scores obtained for ten questions assessing the 5 principles of dialogic public relations. These five principles express different aspects of how organizations must engage in dialog with their targeted public. Each question measures the level of agreement (on a five; point Likert scale) with [illegible] statements related to the five principles of dialogical public relations: mutuality, propinquity, empathy, risk and commitment.
b. Study included logging data In August and September of each year between 2005 and 2009, the first 5000 computer session logs in a computer laboratory were analysed to deter[illegible] what Internet resources were being used by students
c. 620 computer sessions searching for biomedical information were analyzed)
d. The aim of this study was to determine how often the English Wikipedia appears among the top search engine results for health; related queries: A) different sets of keywords wer[illegible] searched to determine the ranking of Wikipedia in Google, Google UK, Yahoo, MSN: 1; 1726 keywords from MedlinePlus index; 2; 966 keywords from the NHS Direct Online inde[illegible] 1,173 keywords from the U.S. National Organization of Rare Diseases index; B) Wikipedia and MedlinePlus Page view statistics were compared for the 20 most visited MedlinePlus Topic and Encyclopedia pages
e. 2 studies were performed: 1) Four search engines (Bing, Yahoo, Google.com, Google.ca) were searched to determine the most common website returned for a list of the most dispensed generic and brand name drugs in the USA (n=278); 2) the number of unique Wikipedia page hits was determined for all study drugs
