# Supplementary material for: Wikis and Collaborative Writing Applications in Health Care: A Scoping Review
Source: J Med Internet Res. 2013 Oct 8;15(10):e210. doi: 10.2196/jmir.2787 (PMC3929050; doi:10.2196/jmir.2787)
Supplement: Supplementary file 3 [file jmir_v15i10e210_app3.pdf]

Authors (Year of publication) G=Grey literature	Who the info is destined to	Purpose	Method to assess quality	Results	Conclusion (of the authors of the paper)	Date of quality assessment	Vote counting based on authors' conclusions*
Aldairy (2011) [133]	Patients	To evaluate 25 orthognathic and jaw surgery information pages including Wikipedia	A validated instrument was used (DISCERN instrument)	Wikipedia achieved the highest score (64/80), and the majority of websites fell well below the maximum score	By directing patients to validated websites, clinicians can ensure patients find appropriate information; however, further development of websites relating to orthognathic surgery is required.	2010 (May)	2
Ayes (2010) [190]	Consumers, healthcare professionals	To evaluate Wikipedia content on toxicology information	A locally-developed grading scale was used	Wikipedia did not provide significant information on toxicology and was not significantly updated or corrected.	Wikipedia is not a good source of information for toxicology as it contains limited information on toxicology and sometimes erroneous information.	2009 and 2010	3
Clauson (2008) [61]	Consumers	To compare the scope, completeness, and accuracy of drug information in Wikipedia with that of a free, online, traditionally edited database (Medscape Drug Reference)	A questionnaire developed by the authors. Scope was measured by the presence or not of an answer (% of presence of answers) and by number of omission errors. Completeness was measured as the percentage of answers that were complete (% of complete answers). Accuracy was measured by the number of inaccurate answers.	Scope: Wikipedia (40.0%) vs MDR (82.5%; $p < 0.001$). Dosing questions: Wikipedia (0%) vs MDR (90.0%). Errors of omission: Wikipedia (48) vs MDR (14). Completeness: Wikipedia (76.0%) vs MDR (95.5%) in MDR ($p < 0.001$). Wikipedia improved over time, as current entries were superior to those 90 days prior ($p = 0.024$). Accuracy (inaccurate answers): Wikipedia (0) vs MDR (4).	Wikipedia provided factually accurate drug information, but it was incomplete, much more likely to contain errors of omission, and thus, of more limited scope than the information available in MDR.	2008 (March)	2
Czarnecka-Kujawa (2008) [134] (G)	Consumers	To assess the comprehensiveness, reliability and readability of Wikipedia's gastroenterology articles	World Health Organization ICD-10 and ICD-9 diagnostic codes in gastroenterology were correlated to articles on Wikipedia on the same topic. Reliability was measured by the number of peer reviewed articles	Comprehensiveness: Of the 203 ICD-10 gastroenterology diagnostic codes: 168 (82.8%) had Wikipedia entries. Of the 178 ICD-9 gastroenterology diagnostic codes, 148 (83.1%) had Wikipedia entries. Reliability: 58/103 (56.3%) articles in Wikipedia were substantiated with at least one peer-reviewed reference. The average number of references per article was 6.8. Readability:	Wikipedia's content in gastroenterology is comprehensive. The reliability is moderate. The articles are readable with a grade level of slightly higher than high school	N/A	2

			referenced per article. Readability was measured with the Flesch-Kincaid readability test and four other standardized tests.	Median Flesch-Kincaid grade level: 13.7; Mean Flesch reading ease score: 32.1; Median automated readability index: 14.9			
Devgan (2007) [58]	Consumers	To examine the quality of Wikipedia articles about the 39 most commonly performed surgical procedures in the USA	Two reviewers reviewed Wikipedia articles using self-developed quantitative metrics of quality	Wikipedia indexed 35 (89.7%) of the most commonly performed surgical procedures. Appropriate articles for patients: 30 (85.7%) Percentage of articles with accurate information: 100% (n=35). Percentage of articles without critical omissions: 62.9% (n=22). Wikipedia articles addressed procedure indications (97.1%, n=34), while 62.9% (n=22) discussed risks. There was a correlation between an entry's quality and how often it was edited.	Wikipedia is an accurate though often incomplete medical reference. Participation of medical and surgical professionals in authoring Wikipedia entries may improve their quality and keep patients more informed.	N/A	2
Dobrogowska-Schlebusch (2009) [99]	Physicians, consumers, patients, healthcare professionals, medical and health students, scientists, medical librarians	To study the quality of 52 medical wikis and the correlation between the quality of wikis and the presence of a quality assurance system, the number of users and the type of software	Criteria created by the Health Summit Working Group (HSWG). All results are pooled (% represents the percentage of the 52 wikis that respected the quality criteria; except for the "links" category that had 23 wikis that could be assessed). Wikis were considered: very good if more than 75% of the HSWG criteria were met and good if between 65 and 75% of HSWG were met.1	Website Design (availability: 98%; navigation: 87%; searchability: 100%) Interactivity: 85% Credibility (source: 48%; update: 92%; correspondence 96%; reviewing procedure: 25%) Content (reliability: 46%; presence of legal notice: 54%) About this site (purpose of page: 85%; privacy policy: 33%) Links (selection: 74%; structure: 83%; content: 83%; backlinks: 40%) Disclaimer (advertising policy: 27%). Among the surveyed Wiki, 18 (35%) had a very good quality, and 10 (19%) were good. Among the 13 of the surveyed wikis that met the "reviewing procedure" criteria, 11 were characterized by very good quality.	Wikis can be an appropriate tool to build a medical or health information source, but only 54% of the reviewed wikis were of good or very good quality as measured by the HSWG criteria. Higher quality scores could be associated with a "reviewing procedure" to control access to certain authors. Wikis peer reviewed and moderated by experts are of better quality than those generated and published by the community of all Internet users.	2008 (August) to 2009 (April)	2
Friedlin (2010) [132]	Consumers	To evaluate the degree of medical knowledge and accuracy of information contained in Wikipedia.	Compare the amount of identical matches to the LOINC (The logical observation identifiers names and codes) database and Wikipedia entries for a random sample of 100 matches.	Of the 1705 parts queried, 1314 matching articles were found in Wikipedia. Of these, 1299 (98.9%) were perfect matches that exactly described the LOINC part.	Wikipedia contains a large amount of scientific and medical data and could effectively be used as an initial knowledge base for specific medical informatics and research projects.	N/A	1
Haigh (2011)	Nursing students	To assess the quality of	Citation tracking for the selected Wikipedia	In total 1473 (56%) of the references cited on the Wikipedia articles	Wikipedia citations should be treated with some caution, but	N/A	2

[136]		references of a random sample of Wikipedia articles.	articles (n=132) were assessed using the typology developed by the British Department of Health in evaluating evidence.	reviewed could be argued to come from clearly identifiable reputable sources. This translates to a mean number of reputable sources of 29 per Wikipedia article.	Wikipedia does have a role to play as a source of health related evidence, and as a useful tool in the teaching of critical appraisal and literature searching. It's use by nursing students when researching information to contribute to assignments should not necessarily be discouraged.		
Hanson (2011) [104]	Dermatologists	To assess the quality of online dermatology resources	Six students assessed the quality using the Silbert criteria. Top-rated resources were defined as a having a Silbert score of 13/20 or higher.	Wikipedia ranked in the top quality dermatology resources (Silbert score not available).	Cutting-edge online dermatology resources (including Wikipedia) represent excellent sources for continuing education for students and clinicians alike. Resources such as these likely represent the future of medical education, as they allow for self-directed and supplementary education as well as remote access.	2011 (July)	1
Hickerson (2009) [122] (G)	Consumers	To determine the perceived value of Wikipedia and WikiHealth compared to 15 non-wiki sites (eg, eMedicine and WebMD)	An online survey was performed with users of Wikipedia (n=45) and WikiHealth (n=16) to determine the perceived value of these wikis compared to the value of 15 non-wiki sites they used (eg, eMedicine and WebMD).	Information from Wikipedia and WikiHealth was perceived just as valuable as non-wiki website information. The overall dialogic scores for the two wikis were also found to have positive and significant correlations to finding the website valuable.[a]	Although a positive correlation exists between the dialogical aspect of wiki pages and perceived value, there remain questions that need further research to understand why the perceived value of wikis is not higher than the perceived value of non-wiki pages.	2 months (year unknown)	2
Johnson (2008) [131]	Students (Primary Care and Internal Medicine)	To compare the utility and efficiency of Google with other medical and nonmedical web-based resources for identifying specific medical information	Students (N=89) used Google and other web-based sources to find answers for a 10-question multiple choice exam. Efficiency was measured by the number of links to find an answer.	Wikipedia showed good answers for 96% (44 of 46 questions). It ranked second in terms of efficiency to find an answer compared to 6 other resources.	No conclusion concerning Wikipedia	2007 (January)	N/A
Judd (2011) [124]	Medical students	To determine medical student's (2nd and 3rd year) perception of the usefulness and reliability of 5	Medical students (n=502) were asked to grade the usefulness and reliability (five-point Likert scale) of Google, Wikipedia, the	Among the 5 websites, Wikipedia was graded 3rd in usefulness (3.99) (after Google (4.38) and eMedicine (4.18)). Wikipedia ranked last in terms of reliability (3.26).	Students' reliance on and familiarity with known tools such and Google and Wikipedia may be contributing to underlying information literacy problems, effectively	2007 (May)	3

		different websites	University of Melbourne Library, eMedicine and The National Institutes of Health[b]		creating a barrier to the development of new information-seeking skills.		
Kim (2010) [54]	Pathology residents	To evaluate Wikipedia content about pathology informatics	Items from students' recognized curriculum (vida supra) were compared to the students' curriculum and assessed quality by using five-point Likert scales. Five topics were assessed.	Up-to-date: 4.18; Quality: 4.08; Completeness: 4.05; Appropriate for advanced learners: 3.93; Appropriate for beginners 3.85.	Wikipedia is a comprehensive, high quality, current and appropriate for beginners and advanced learners.	2010 (March)	1
Lavsa<br><br>(2011) [135]	Students (pharmacy)	To assess the accuracy and completeness and referencing of the Wikipedia pages for the 20 top prescribed medications in the USA.	Four pharmacists independently assessed the articles for specific categories of information typically found in medication package inserts. Wikipedia articles were compared to package inserts, Micromedex Drugdex Evaluations, Clinical Pharmacology, and Lexi-Comp. Each article was evaluated for the presence of each category, and for each category that was present, the information was designated as accurate (no discrepancies from FDA labeling), complete (contain all subcategories), and referenced (fully, partially, or none).	Information categories most frequently absent were drug interactions and medication use in breastfeeding. Information on contraindications and precautions, drug absorption, and adverse drug events was most frequently found to be inaccurate. Descriptions of off-label indications, contraindications and precautions, drug interactions, adverse drug events, and dosing were most frequently incomplete. Referencing was poor across all articles.	Variability in the content, accuracy, completeness, and referencing of drug information in Wikipedia was found. Students should not use Wikipedia sources for referencing and it should not be used for patient care because of the potential for patient harm resulting from incomplete or inaccurate information.	N/A	3
Leithner (2010) [195]	Consumers	To assess the scope, completeness, and accuracy of information found on osteosarcoma in Wikipedia compared to the patient version and the health	Using a questionnaire comprised of 20 questions, two surgeons and a medical student assessed the scope, completeness, and accuracy of information on osteosarcoma from	NCI professional version: 50/60 points; NCI Patient version: 40/60 points. Wikipedia: 33/60 points. There was only a statistically significant difference between the NCI professional version and Wikipedia p=0.039). All three reviewers preferred Wikipedia when asked for the ease of use to find	The quality of osteosarcoma-related information found in the English Wikipedia is good but inferior to the patient information provided by the NCI. Wikipedia should include links to more definitive sources, such as those maintained by the NCI and	2009 (April)	2

		professional version of the US National Cancer Institute (NCI) website.	Wikipedia, the patient version and the health professional version of the National Cancer Institute's (NCI) website. The answers to the 20 questions were verified with authoritative resources and international guidelines.	patient-related information.	professional international organizations. Frequent checks should make sure such external links are to the highest quality and to the best-maintained aggregate sites on a given healthcare topic.		
Lorenz (2010) [183]	Consumers and dentists	To assess the scientific quality of 265 articles on dentistry (German Wikipedia)	Wikipedia content was subjectively compared to recognized scientific sources (textbook, scientific articles, guidelines) and classified as "acceptable", "partially acceptable" and "inacceptable" for a textbook.	74 articles (28,4%): "appropriate for a medical textbook"; 146 (55,9%) articles: "partially qualified»; 41 (16%) articles: "not qualified". 220/261 (84,3%) articles on dentistry were appropriate for patient information; 123/261 articles (47,1%) presented a complete presentation of the topic.The quality of an article decreased with having more edits and editors.	Individuals interested in dental topics should not exclusively rely on Wikipedia. Increasing peer-review by experts might improve quality.	2008 (January)	2
McInnes (2011) [182]	Consumers	To calculate the readability of websites on various diseases.	The names of 22 health conditions were entered into five search engines, and the readability of the first 10 results for each search were evaluated using Gunning FOG, SMOG, Flesch-Kincaid and Flesch Reading Ease tests.	Wikipedia articles (Reading Grade (RG)=15.21, 95% CI=14.44–15.99) were significantly harder to read than other .org websites (p<.001). Wikipedia articles were even more difficult to read than .edu sites and had a FRE score of 31.22 (95% CI=27.96–34.48) (a score close to being considered 'very difficult')	Some of the most frequent search results (such as Wikipedia pages) were amongst the hardest to read. Health professionals, with the help of public and specialised libraries, need to create and direct patients towards high-quality, plain language health information in multiple languages.	2010 (January)	3
Mercer (2007) [196]	Consumers	To review Wikipedia's handling of mental health topics	One reviewer subjectively reviewed three articles (autism, bipolar disorders, reactive attachment disorder) for obvious errors or omissions.	The included mental health pages had missing information and poor discussions.	A higher quality of information would be desirable in a source so easily accessible.	2006 (July)	3
Mühlhauser (2008) [197]	Consumers	To compare the quality of health information provided by the German Wikipedia and 2 German health insurances	22 students investigated 1 topic and were reviewed by one of the co-authors. A checklist (47 evidence based items) was used.	Results for the 21 topics in Wikipedia (987 items): Correct/fulfilled: 18% (n=174); Wrong/Not fulfilled: 3% (n=32); Incomplete/Partially fulfilled: 137(14%); Missing: 59% (n=582); Not applicable: 6% (n=62)	Neither German Wikipedia nor 2 major German health insurances provide medical information that sufficiently fulfils internationally agreed criteria for evidence based patient or consumer	2007 (December)	3

		websites.			information.		
Pender (2009) [63]	Students (medicine)	To address the suitability of Wikipedia as a source for medical students	One expert compared de-identified copies of Wikipedia, eMedicine, AccessMedicine and UpToDate articles using a self-developed scale to rank accuracy, coverage, concision, currency and suitability of the resources. Accessibility and usability were assessed by medical librarians.	The entries in Wikipedia, in comparison with the other resources, were easy to access, navigate and well presented. Although reasonably concise and current, the Wikipedia entries failed to cover key aspects of two of the topics, and contained some factual errors.	Wikipedia was found to be very accessible but was judged unsuitable for medical students to base their learning on.	N/A	3
Rajagopalan (2010) [198]	Consumers	To assess the quality of online cancer information on Wikipedia and the website of the National Cancer Institute's physician Data Query (NCI PDQ)	Information of the sources was compared to textbook information. Reliability (inter-observer variability and test-retest reproducibility), readability (calculated from word and sentence length) and accuracy (locally developed scoring system) were assessed.	No differences in the combined depth and accuracy of content between Wikipedia and NCI PDQ. Controversial aspects of cancer care were poorly discussed in both sources. Wikipedia was significantly less readable than NCI PDQ (Flesch-Kincaid Grade Level score: 9.6 (SD 1.5) vs. 14.1 (SD 0.5) ($p < 0.0001$)).	Wikipedia and NCI PDQ entries have a comparable depth and accuracy but Wikipedia was less readable.	2009 (August)	2
Schweitzer (2008) [121]	Psychology (undergraduate students)	To assess Wikipedia's coverage of psychology-related concepts	No quality assessment method (study only assessed breadth of coverage). One author selected 100 psychology topics to review.	Among the 100 topics 81 were covered in Wikipedia.	The results demonstrated that Wikipedia's coverage of psychological topics was comprehensive and prominently displayed on the major search engines.	N/A	N/A
Tulbert (2011) [199]	Consumers	To assess the readability and length in words of online patient education materials	Online patient information was compared to information produced by the American Academy of Dermatology using the Flesch-Kincaid Grade Level (FKGL) and Flesch Reading Ease (FRE) tests.	Wikipedia proved significantly harder to comprehend than all other sources. None of the Wikipedia articles was below the ninth grade level, and 11 of 15 Wikipedia articles exceeded 12.0 on the FKGL. No articles from Wikipedia exceeded a FRE score of 60.0.	No single source of commonly used internet patient-education material demonstrates optimal features with regard to readability, length, and presence of photographic illustrations. Wikipedia should use professional editors to increase readability of articles destined for patient education.	2009 (April)	2
Wood (2010)	Pathology	To compare the content of	One pathologist trainee reviewed the content of	The entries were generally informative, accurate,	Wikipedia is an informative and accurate source for	2009	2

[137]	students	Wikipedia to that of the Kumar et al Pathological basis of disease (8th ed) textbook	16 Wikipedia articles relating to different pathologies and subjectively compared them to the Kumar textbook content	comprehensive and useful resources for medical education. The entries are generally well referenced and provide external links to other good resources.	pathology education, particularly if used in combination with other learning materials. Caution must be advised with regards to the medical information presented.	(December)	
Wu (2010) [96] (G)	Consumers	To compare the readability and six specific text features of Wikipedia and Knol.	Wikipedia articles and Google Knol pages were compared in different fields of which: Alzheimer's disease, autism, Helicobacter pylori, influenza, lung cancer, multiple sclerosis, subarachnoid hemorrhage and tuberculosis. Six features were compared: page views per year, text words, readability, page strength, citation numbers, and citation types. Readability was assessed using: Gunning-Fog Score, Flesch Kincaid Reading Ease, Flesch Kincaid Grade Level, SMOG Index, Coleman Liau Index, and Automated Readability Index	Number of text words: no significant difference (p=0.327); Page views per year: Wikipedia: 145264.1 Knol 10144.5; p=0.12; Page ranking in search engines: Wikipedia: 0.629 vs. Knol 0.243 p=0.012; Citation numbers (all citations included): Wikipedia: 141 vs Knol 17 p=0.12; Readability: Wikipedia 8.5 Knol 9.875 p=0.048 (dwikipedia is easier to read); Citation of journals: Wikipedia: 8.602 vs Knol: 12.372; p= 0.042. Folk-oriented Wikipedia has better popularity, influence, ranking ability, and amount of references. Expert-oriented Knol provides more difficult articles and cites more authority resources. To improve Wikipedia, it needs to control citation numbers and cite more authoritative resources to increase accuracy and credibility. For Knol, it needs to do search engine optimization. Wikipedia AND Knol need to increase its readability to fit much more general readers	The result can help online encyclopedia's improve their quality from different viewpoints. In addition, users could choose appropriate articles from different valuable resources based on our investigations. The researchers have to conduct further studies with consideration of these shortcomings.	N/A	N/A

* 1: Mostly a reliable source, high quality info; 2: Partially reliable: e.g. needs improvement or updates; 3: Not reliable, should not be used

a. The dialogical score is a composite score based on a summary of the scores obtained for ten questions assessing the 5 principles of dialogic public relations. These five principles express different aspe of how organizations must engage in dialog with their targeted public. Each question measures the level of agreement (on a five-point Likert scale) with 5 statements related to the five principles of dialogical public relations: mutuality, propinquity, empathy, risk and commitment.
b. This was a survey and 92% response rate among 549 surveyed.
