# Supplementary material for: Wikis and Collaborative Writing Applications in Health Care: A Scoping Review
Source: J Med Internet Res. 2013 Oct 8;15(10):e210. doi: 10.2196/jmir.2787 (PMC3929050; doi:10.2196/jmir.2787)
Supplement: Supplementary file 4 [file jmir_v15i10e210_app4.pdf]

Author (Year)	Study design	Type of CWA (software)	Participants and context of study (field of study)	Intervention	Comparison	Main results
Ioannis (2011) [107]*	Clinical trial**	Google Docs (Google Docs)	Patients (n=80) undergoing a 6-month secondary prevention program after an acute coronary syndrome (Cardiology)	Using an online diary, participants (n=35) had to log on at least once a month to enter their blood pressure measurements and minutes of physical activity. They had access to a Google Doc where they could find instructions from a physician who analyzed their online diary.	Standard care (n=45)	The online support program realized significant changes to their blood pressure, cholesterol levels and smoking status compared with the standard care group but there was no statistically significant difference for physical activity.
Phadtare (2009) [27]	RCT***	Google Docs (Google Docs)	2nd and 3rd year medical, physiotherapy and nursing students (n=48) enrolled in a scientific writing course had to write a scientific paper in groups of two.<br><br>(Medical education)	Pairs (n=12) of students used Google Docs to write, review, edit and share their manuscripts online with their mentors who gave them feedback	Pairs (n=12) of students used standard, computer-based word processing software and contacted their mentors by phone for feedback	Google Docs group had better overall manuscript quality (mean Six-Subgroup Quality Scale (SSQS) score (SD) = 75.3 (14.21) vs. control group (mean SSQS (SD) = 47.27 (14.64) (p = 0.0017). Participant satisfaction (SD) was higher in the Google Docs group (4.3 (0.73) vs. control group (3.09 (1.11)) (p = 0.001) (5-point Likert scale). Control group had fewer communication events (SD) with their mentors vs. Google Docs group (0.91 (0.81) vs. 2.05 (1.23) (p=0.0219)
Stutsky (2009) [109]**** (D)	RCT	Wiki (Platinum version *Peanut Butter Wiki* (PBwiki))	Nurse educators (n=51) participated in a 12-week online learning community with a wiki where storytelling was shared to develop nursing leadership practices. (Nursing)	Participants (n=26)***** had access to a wiki within a moderated learning community. The facilitator organized the wiki pages, posted stories, and assisted nurse educators in analyzing their own leadership stories	Participants (n=25)***** had access to a wiki without a moderated learning community (self-organizing	There were no differences between the communities, except on the teaching presence subscale of direct instruction, where the facilitated community was rated significantly higher. Nurse educators in both communities significantly increased their own perceived leadership practices (The Leadership Practices Inventory) and

					community). In the self-organizing community, community members were required to analyze their own stories and share their stories with others.	perceived levels of empowerment (structural (Conditions of Work Effectiveness Questionnaire-II) and psychologic (Psychological Empowerment Instrument). Educators in both learning communities identified that their communities included the elements of teaching, cognitive, and social presence (Community of Inquiry Instrument). Given increases in empowerment levels, it was determined that both online learning communities could be considered empowering environments.
Moeller (2010) [108]	RCT	Wiki (not available)	Medical students (n = 237) participating in a problem-based learning (PBL) course were enrolled in a multiple arm study comparing students (n=99) in a classical PBL (cPBL) course to students (n=138) participating in a blended PBL (bPBL) course involving different elearning tools including a wiki, a chat and an interatice diagnostic context. (Medical education)	bPBL with wiki support	bPBL without wiki support (NB: more than one control existed in this study. other controls: cPBL, bPBL with chat support, bPBL with an interactive diagnostic context	Self-administered questionnaires revealed the following results: 1- Learning effect: wikis significantly reduced the perception of PBL case difficulty compared to chat and interactive diagnostic context. (No difference of wikis on other aspects: acceptance of the case's difficulty; feeling to have covered everything; preparedness for the exam; right diagnosis; number of right answers in the self-test; 2- Knowledge acquisition: perceived increased from pre to post significantly for all wiki groups. 3- Communication: wikis improved the perceived time to communicate, organization of work flow via communication, the density of communication, and the longevity of information communicated. 4- Collaboration: No significant perceived differences between wikis and other PBL groups; however, Chat improved perceived

collaboration significantly. 5- Satisfaction: significantly increased with the bPBL with wiki support. 6- Wiki groups show significantly lower diagnostic selectivity; lower knowledge about diagnostic costs and lower knowledge about adequate diagnostic steps. 7- Wikis support 3 of the 7 steps in PBL: hypothesis formation; documentation of results; and working on tasks

* This paper is an abstract. No full text could be identified

** Methods for this abstract are very poorly described. It is impossible to determine if the paper was randomized or not.

*** RCT: randomized controlled trial

**** This paper is a PhD. dissertation.

***** In the end, some participants dropped out of the project and left 19 participants in the facilitated group compared to 16 in the self-organizing group.
